# Using a reaction‐diffusion model to estimate day respiration and reassimilation of (photo)respired CO_2_ in leaves

**DOI:** 10.1111/nph.15857

**Published:** 2019-05-11

**Authors:** Herman N. C. Berghuijs, Xinyou Yin, Q. Tri Ho, Moges A. Retta, Bart M. Nicolaï, Paul C. Struik

**Affiliations:** ^1^ Centre for Crop Systems Analysis Wageningen University & Research Droevendaalsesteeg 1 6708 PB Wageningen the Netherlands; ^2^ Flanders Center of Postharvest Technology/BIOSYST‐MeBioS Katholieke Universiteit Leuven Willem de Croylaan 42 Leuven B‐3001 Belgium; ^3^ Department of Crop Production Ecology Swedish University of Agricultural Sciences Ulls väg 16 Uppsala 75651 Sweden; ^4^ Food Chemistry & Technology Department Teagasc Food Research Centre Moorepark, Fermoy, Co. Cork P61 C996 Ireland

**Keywords:** C_3_ photosynthesis, mesophyll conductance, photorespiration, reaction‐diffusion model, reassimilation, respiration

## Abstract

- Methods using gas exchange measurements to estimate respiration in the light (day respiration $R_{d}$) make implicit assumptions about reassimilation of (photo)respired CO_2_; however, this reassimilation depends on the positions of mitochondria.
- We used a reaction‐diffusion model without making these assumptions to analyse datasets on gas exchange, chlorophyll fluorescence and anatomy for tomato leaves. We investigated how $R_{d}$ values obtained by the Kok and the Yin methods are affected by these assumptions and how those by the Laisk method are affected by the positions of mitochondria.
- The Kok method always underestimated $R_{d}$. Estimates of $R_{d}$ by the Yin method and by the reaction‐diffusion model agreed only for nonphotorespiratory conditions. Both the Yin and Kok methods ignore reassimilation of (photo)respired CO_2_, and thus underestimated $R_{d}$ for photorespiratory conditions, but this was less so in the Yin than in the Kok method. Estimates by the Laisk method were affected by assumed positions of mitochondria. It did not work if mitochondria were in the cytosol between the plasmamembrane and the chloroplast envelope. However, mitochondria were found to be most likely between the tonoplast and chloroplasts.
- Our reaction‐diffusion model effectively estimates $R_{d}$, enlightens the dependence of $R_{d}$ estimates on reassimilation and clarifies (dis)advantages of existing methods.

## Introduction

Quantifying respiration is important for accurately predicting net ecosystem productivity, as respiratory losses can account for ≤ 40% of gross primary production (Gifford, 2003). At the leaf level, respiration in the light, also called ‘day respiration’ ($R_{d}$), is an important term in the model of Farquhar, von Caemmerer & Berry (‘FvCB model’; Farquhar *et al*., 1980), which is used widely as the basic model component for predicting ecosystem productivity. Unlike leaf respiration in the dark ($R_{\mathrm{dk}}$), day respiration occurs simultaneously with photosynthetic CO_2_‐assimilation and is difficult to determine by gas‐exchange measuring systems. Uncertainties have arisen over years with regard to, for example, whether $R_{d}$ differs from $R_{\mathrm{dk}}$ and whether $R_{d}$ varies with different conditions (Griffin & Turnbull, 2013). Efforts continued recently in updating the biochemical processes underlying $R_{d}$ (Tcherkez *et al*., 2017a,2017b;) and in how to better measure or quantify this parameter (Buckley *et al*., 2017; Farquhar & Busch, 2017; Tcherkez *et al*., 2017a,2017b; Gong *et al*., 2018; Way *et al*., 2019).

Techniques have been developed to measure $R_{d}$ directly (Loreto *et al*., 1999, 2001; Pärnik & Keerberg, 2007; Gong *et al*., 2015; Tcherkez *et al*., 2017a; Gauthier *et al*., 2018), but these techniques usually require access to sophisticated and expensive isotope discrimination measuring devices and are often unavailable. Methods exist to indirectly estimate $R_{d}$ in C_3_ leaves from conventional gas‐exchange measurements (Kok, 1948; Laisk, 1977; Brooks & Farquhar, 1985), sometimes combined with chlorophyll fluorescence measurements (Yin *et al*., 2009). The Laisk method (Laisk, 1977; Brooks & Farquhar, 1985) has become the most common one. It explores the linear part of several $A_{N}-C_{i}$ curves at low *C*_i_ concentrations ($C_{i}$ is the intercellular CO_2_ partial pressure), measured at difference irradiances. The negative net CO_2_ assimilation rate $A_{N}$ at the point at which the linear $A_{N}-C_{i}$ curves intersect is the estimated $R_{d}$. $C_{i}$ at the intersection point ($C_{i}^{*}$) is often used as the CO_2_ compensation point $\Gamma^{*}$, at which the amount of CO_2_ produced by photorespiration equals the amount of CO_2_ consumed by ribulose biphosphate (RuBP) carboxylation. The theoretical basis of the Laisk method is the FvCB model:

(Eqn 1) $$A=\frac{\left(C_{c}-\Gamma^{*}\right)X_{1}}{C_{c}+X_{2}}-R_{d},$$

where $C_{c}$, is the CO_2_ partial pressure at the carboxylating sites of Rubisco. The terms $X_{1}$ and $X_{2}$ depend on whether carboxylation is limited by Rubisco activity, electron transport (Farquhar *et al*., 1980), or triose phosphate utilization (Sharkey, 1985). If it is limited by electron transport, $X_{1}$ is a function of incident irradiance ($I_{\mathrm{inc}}$) and Eqn (Eqn 1) can generate the Laisk plot. However, theoretically, the Laisk method works only if $C_{c}=C_{i}$. This was a common assumption at the time when the method was proposed as mesophyll resistance (*r*_m_) was then believed to be negligible compared with stomatal resistance to CO_2_ transfer (*r*_s_). Nowadays, *r*_m_ (and its inverse, mesophyll conductance *g*_m_) is proven to be relevant under a wide range of conditions and across different species (Evans *et al*., 1986; Flexas *et al*., 2008; Niinemets *et al*., 2009). Consequently, there is a large CO_2_ gradient between the intercellular spaces and the chloroplasts (Von Caemmerer & Evans, 1991; Von Caemmerer *et al*., 1994):

(Eqn 2) $$C_{c}=C_{i}-\frac{A_{N}}{g_{m}}.$$

Combining the nonlinear Eqn (Eqn 1) and the linear Eqn (Eqn 2) results in a solution in which $A_{N}-C_{i}$ curves at different irradiances will not necessarily intersect at the same value of the $A_{N}$ axis. Tholen *et al*. (2012) pointed out that the method of calculating *g*_m_ according to Eqn (Eqn 2) implicitly assumes that CO_2_ produced by respiration and photorespiration (which will be called ‘(photo)respired CO_2_’ hereafter), and the CO_2_ molecules from intercellular air‐spaces experience the same mesophyll resistance. However, (photo)respired CO_2_, if being reassimilated, probably experiences the chloroplast resistance component (*r*_ch_) only. By contrast, the CO_2_ molecules from intercellular air‐spaces experience cell wall and plasma‐membrane resistance (*r*_wp_) as well as *r*_ch_ (the sum of *r*_wp_ and *r*_ch_ makes the total *r*_m_). Therefore, Tholen *et al*. (2012) concluded that $g_{m}$ as defined by Eqn (Eqn 2), is an apparent parameter. They demonstrated that if this scheme for *r*_m_ resistance components is considered, there is no guarantee that *A*_N_–*C*_i_ curves at different $I_{\mathrm{inc}}$ will intersect at the same *C*_i_ and at the same $A_{N}$. This implies that $R_{d}$ estimated by the Laisk method may depend on the resistance scheme, which, in turn, depends on the cellular position of (photo)respired CO_2_ release (see later in this Introduction). Moreover, as pointed out by Yin *et al*. (2011), the Laisk method has the practical problem that all measurements are at lower than ambient‐air CO_2_ concentrations (*C*_a_), requiring the correction of gas exchange data for CO_2_ leakage (Flexas *et al*., 2007).

An alternative method to estimate $R_{d}$ is the Kok method (Kok, 1948). This method exploits the fact that the response of $A_{N}$ to irradiance is approximately linear at low irradiances. $R_{d}$ is calculated as the intercept of this linear relationship. However, at irradiances close to the light compensation point or lower, this slope may become steeper (Kok, 1948; Farquhar & Busch, 2017; Tcherkez *et al*., 2017a). In order to avoid this so‐called Kok effect, irradiances under which this method is applied should be above this breakpoint (Brooks & Farquhar, 1985). If evaluated from the electron‐transport limited form of Eqn (Eqn 1), the Kok method actually assumes that the quantum yield of Photosystem II electron transport ($\Phi_{2}$) is constant over the same range of irradiances. However, $\Phi_{2}$ has been observed to decline with increasing irradiances (Genty & Harbinson, 1996) even under low‐irradiance conditions (Yin *et al*., 2011, 2014). To account for this decline, Yin *et al*. (2009) proposed a method which also exploits the $A_{N}-I_{\mathrm{inc}}$ curve at low irradiance, but combines it with simultaneously measured chlorophyll fluorescence to assess $\Phi_{2}$, and $R_{d}$ is estimated as the intercept of the linear regression of $A_{N}$ vs $\Phi_{2}I_{\mathrm{inc}}/4$. To distinguish it from the Kok method, it has been called the Yin method (Tcherkez *et al*., 2017b). Theoretically, both Kok and Yin methods work only for nonphotorespiratory conditions (Yin *et al*., 2011), or for photorespiratory conditions if *C*_c_ is made to be constant across irradiance intensities. They are sometimes also used for photorespiratory conditions where *C*_c_ varies, because usually the plot of measured $A_{N}$ vs *I*_inc_ or vs $\Phi_{2}I_{\mathrm{inc}}/4$ seems linear.

However, when the Yin or Kok methods are applied directly to photorespiratory conditions where only $C_{a}$ is controlled, the problem associated with the variation of *C*_i_ or *C*_c_ with $I_{\mathrm{inc}}$ may become relevant. This is because linear regression of $A_{N}$ against $I_{\mathrm{inc}}$ (Kok method) or against $\Phi_{2}I_{\mathrm{inc}}/4$ (Yin method) implicitly assumes that *C*_c_ does not vary with $I_{\mathrm{inc}}$ within the data range used. The present understanding of stomatal conductance (*g*_s_) and *g*_m_ shows that *g*_s_ and *g*_m_ can have very low values at low $I_{\mathrm{inc}}$; when combined with the FvCB model, the low *g*_s_ and *g*_m_ values predict that *C*_c_ decreases sharply with increasing $I_{\mathrm{inc}}$ within the low $I_{\mathrm{inc}}$ range (Farquhar & Busch, 2017). It is known also that a combined FvCB and conductance model can implicitly account for reassimilation of (photo)respired CO_2_ (Von Caemmerer, 2013). Therefore, both the Kok and Yin methods, when applied to photorespiratory conditions, may implicitly assume that there is no reassimilation of (photo)respired CO_2_ as they assume that *C*_c_ remains constant under a range of low light intensities. In fact, there is both experimental (Loreto *et al*., 1999; Haupt‐Herting *et al*., 2001; Pärnik & Keerberg, 2007; Busch *et al*., 2013) and theoretical (Tholen *et al*., 2012; Ho *et al*., 2016; Berghuijs *et al*., 2017; Yin & Struik, 2017) evidence that a substantial fraction of the (photo)respired CO_2_ is used for RuBP carboxylation in the chloroplasts before it can escape to the atmosphere. If recycling of CO_2_ is not accounted for to determine $R_{d}$, the true $R_{d}$ is possibly underestimated (Loreto *et al*., 1999; Gong *et al*., 2018). Instead of using simple linear regressions, using the combined FvCB and *g*_m_ model to fit types of experimental data (that each method relies on) under photorespiratory conditions would give an estimation of *R*_d_ while simultaneously considering reassimilation. However, *r*_m_, let alone its components, *r*_wp_ and *r*_ch_, is not known beforehand. In fact, an estimation of *r*_m_ or *g*_m_ would require an estimate of *R*_d_ beforehand (Harley *et al*., 1992; Yin & Struik, 2009).

According to the resistance model of Tholen *et al*. (2012), the fraction of reassimilation of (photo)respired CO_2_ depends on the relative magnitude of individual resistance components along the path from leaf surface to Rubisco carboxylation sites. These resistances include *r*_s_, *r*_wp_, *r*_ch_ and *r*_cx_, where *r*_cx_ is carboxylation resistance that can be expressed from Eqn (Eqn 1) as $(C_{c}+X_{2})/X_{1}$ (Tholen *et al*., 2012). Because at least *r*_s_ and *r*_cx_ are known to depend on CO_2_ concentration and irradiance, reassimilation also may be affected by environmental variables. A similar statement can be made for any impact of physiological parameters on reassimilation. Yin & Struik (2017) extended the model of Tholen *et al*. (2012) and pointed out that the fraction of reassimilation of (photo)respired CO_2_ not only depends on the relative magnitude of the resistance components but also on intracellular arrangements of chloroplasts and mitochondria. They highlighted that the impact of such intracellular arrangements of organelles is hard to be dealt with by resistance models when the chloroplast coverage of mesophyll areas is low. The resistance model of Tholen *et al*. (2012) assumes either that there is no CO_2_ gradient in the cytosol (Tholen *et al*., 2014) or that the mitochondria are located in a cytosol layer between the cell wall and the chloroplasts (Berghuijs *et al*., 2015, 2016). In reality, the mitochondria are mostly located between the chloroplasts and the tonoplasts (Hatakeyama & Ueno, 2016), intimately associated with chloroplasts (Sage & Sage, 2009). Previous studies (Berghuijs *et al*., 2017; Xiao & Zhu, 2017; Yin & Struik, 2017) showed that the modelled position of mitochondria relative to the chloroplasts can substantially affect $A_{N}$, reassimilation of photorespired CO_2_ and $g_{m}$.

The CO_2_ diffusion pathway between the intercellular airspaces and the chloroplasts is rather complex. Various methods to estimate *R*_d_ and other physiological parameters of the FvCB model make simplifying assumptions about this pathway. Such simplification results either in not considering the reassimilation of (photo)respired CO_2_ at all or in implicit assumptions about the location of (photo)respired CO_2_ release. This problem can be avoided by using reaction‐diffusion models that describe the CO_2_ diffusion pathway within mesophyll cells in sufficient detail that they do not have to make these implicit assumptions.

Most reaction‐diffusion models for photosynthesis (Tholen & Zhu, 2011; Ho *et al*., 2016; Retta *et al*., 2017; Xiao & Zhu, 2017) are complex and have lengthy computational times. We previously developed a simple reaction‐diffusion model that can be used as an alternative to mesophyll resistance‐based models to estimate photosynthetic parameters (Berghuijs *et al*., 2017). Here we will use this model as a tool to assess whether the Kok method, the Yin method and the Laisk method underestimate *R*_d_ due to their assumptions with regard to reassimilation. Given that reassimilation and mesophyll resistance are affected by the assumed position of the mitochondria relative to the chloroplasts (Berghuijs *et al*., 2017; Xiao & Zhu, 2017), any assumption about the location of mitochondria in mesophyll cells may affect the estimates of the photosynthetic parameters. Therefore, we will also identify the most likely position of the release of (photo)respired CO_2_, relative to the position of the chloroplasts.

## Materials and Methods

### Experimental data

We used published datasets from two experiments (Berghuijs *et al*., 2015; Ho *et al*., 2016), both consisting of simultaneous measurements of gas exchange and chlorophyll fluorescence (Table 1). The Berghuijs *et al*. (2015) dataset contains measurements taken from the distal leaflet from 15‐ and 25‐d‐old leaves from the tomato (*Solanum lycopersicum*) cultivars Admiro, Doloress and Growdena. The Ho *et al*. (2016) dataset contains measurements taken from leaves of the same cultivars as in the experiment of Berghuijs *et al*. (2015). For each cultivar, two types of leaflets were used for measurements. The first was the distal leaflet of the uppermost fully expanded leaf (the ‘upper leaf’). The second was the most distal leaflet from a leaf four layers below the upper leaf (the ‘lower leaf’). In both experiments, gas exchange measurements were taken under photorespiratory and nonphotorespiratory conditions; but leaf anatomical measurements were taken in the experiment of Berghuijs *et al*. (2015) only.

**Table 1:** Overview of the two experimental datasets used in this study

Source:	Berghuijs *et al*. (2015)	Ho *et al*. (2016)
Cultivars:	Admiro, Doloress, Growdena	Admiro, Doloress, Growdena
Leaf types:	15‐d‐old leaves, 25‐d‐old leaves	Upper leaves, lower leaves
Anatomical measurements:	Yes	No
*A* − *C*_a_ curves		
PR	*I*_inc_ = 1500 μmol m^−2^ s^−1^ *O *=* *21 kPa	*I*_inc_ = 1000 μmol m^−2^ s^−1^ *O *=* *21 kPa
NPR	*I*_inc_ = 1500 μmol m^−2^ s^−1^ *O *=* *2 kPa	*I*_inc_ = 1000 μmol m^−2^ s^−1^ *O *=* *2 kPa
*A* − *I*_inc_ curves		
PR	*C*_a_ = 40 Pa *O* = 21 kPa	*C*_a_ = 38 Pa *O* = 21 kPa
NPR	*C*_a_ = 100 Pa *O* = 2 kPa	*C*_a_ = 100 Pa *O* = 2 kPa

PR, photorespiratory conditions; NPR, nonphotorespiratory conditions.

### Mesophyll microstructural model and CO_2_ reaction‐diffusion model

We used measured anatomical properties to parameterize the model for the leaf microstructure of each leaf type (three cultivars × two leaf ages/positions × two datasets = 12 leaf types in total). We used measurements of $t_{\mathrm{wall}}$ (cell wall thickness), $t_{\mathrm{cyt}}$ (cytosol thickness), $t_{\mathrm{str}}$ (stroma thickness), $S_{c}/S_{m}$ (surface area ratio of exposed chloroplasts to exposed mesophyll) and $S_{m}/S$ (surface area of exposed mesophyll to leaf) to parameterize the model for leaf types from the Berghuijs *et al*. (2015) dataset. As the Ho *et al*. (2016) dataset lacks measurements of these parameters, we assumed for each leaf type in this dataset that $t_{\mathrm{wall}}=120\;\mathrm{nm}$, $t_{\mathrm{cyt}}=250\;\mathrm{nm}$, $t_{\mathrm{str}}=2.5\;\mu m$, $S_{c}/S_{m}=0.90$ and $S_{m}/S=16$, which are within the same range of the values of Berghuijs *et al*. (2015). For all types, we assumed that the Michaelis–Menten coefficient for carboxylation by Rubisco, $K_{\mathrm{mC}}$ equals 26.7 Pa (Ho *et al*., 2016), the Michaelis–Menten coefficient for oxygenation by Rubisco, $K_{\mathrm{mO}}$ equals 16.4 kPa (Ho *et al*., 2016), and Rubisco specificity *S*_c_/o = 2.6 kPa Pa^−1^ (Tholen *et al*., 2012). We ran simulations for three different scenarios. (Photo)respired CO_2_ is released either in the inner cytosol (layer between chloroplasts and tonoplast), in cytosol gaps (spaces between two neighbouring chloroplasts) or in the outer cytosol (layer between the chloroplasts and the plasma membrane). Further details on the reconstruction of the leaf geometry, modelling of the scenarios for (photo)respired CO_2_ release, the calculation of the fraction of (photo)respired CO_2_ that is reassimilated, and the reaction‐diffusion model are provided by Berghuijs *et al*. (2017).

### Parameterization and validation of the reaction‐diffusion model

The linear electron transport rate was calculated as $J=sI_{\mathrm{inc}}\Phi_{2}$, where $I_{\mathrm{inc}}$ is the irradiance for each measurement, $\Phi_{2}$ is the quantum yield of Photosystem II, and *s* is a proportionality coefficient, which is calculated as the slope of the linear regression between $A_{N}$ and $I_{\mathrm{inc}}\Phi_{2}/4$ under nonphotorespiratory conditions (Yin *et al*., 2009).

For both datasets, we used only irradiance data ≤ 150 μmol m^−2^ s^−1^, the range usually used for linear regression by the Kok and Yin. We estimated day respiration rate $R_{d}$ with the reaction‐diffusion model by minimizing the sum of the squared residuals of the measured and the simulated CO_2_ assimilation rates. $R_{d}$ was estimated for each scenario for the location of the release of (photo)respired CO_2_. For this optimization, we used the matlab (The Mathwork, Natick, USA) function lsqnonlin(). Supporting Information Notes S1 contains documentation of the source code (Notes S2) for this procedure, with a user guide. The values estimated by the reaction‐diffusion model were compared with the values of $R_{d}$ estimated by the Yin and Kok methods using linear regression on the same experimental data.

The rate of triose phosphate utilization $T_{p}$ was determined as $\left(A_{p}+R_{d}\right)/3$, where $A_{p}$ is the mean observed value at the highest $C_{a}$ of the CO_2_ response curve measured under photorespiratory conditions. The reaction‐diffusion model also was used to estimate $V_{\mathrm{cmax}}$ (maximum rate of Rubisco carboxylation) for each scenario, by minimizing the squared difference between the predicted and the measured $A_{N}$, using only data from the CO_2_ response curve measured for O = 21 kPa and *C*_a_ < 30 Pa. The remaining gas exchange data for each leaf type were used to validate the model.

### Simulations of CO_2_ assimilation under conditions of the Laisk method

For using the reaction‐diffusion model to simulate CO_2_ assimilation under the conditions only at which the Laisk method is applied, we adjusted the boundary condition at the interface of the intercellular airspace, such that $C_{i}$ was used as input for the model, rather than *g*_s_ and $C_{a}$. In line with the assumptions of the Laisk method, we assumed that photosynthesis is limited by electron transport. We simulated CO_2_ response curves at four intensities of *I*_inc_: 150_,_ 100, 50 and 25 μmol m^−2^ s^−1^, using a prefixed value for $\Gamma^{*}$ and the $R_{d}$ estimates obtained by the reaction‐diffusion model as input. From measurements of $\Phi_{2}$ in the light response curve and the estimate of *s*, we calculated the rate of linear electron transport for these irradiances, according to $J=s\Phi_{2}I_{\mathrm{inc}},$ as 52.0, 36.3, 19.3 and 9.1 μmol m^−2^ s^−1^, and used these as input for simulation. We ran these simulations for each of the three scenarios with regard to the location of (photo)respired CO_2_ release. The results were used to investigate how these scenarios would affect the estimates of *R*_d_ if the Laisk method is applied to similar experimental conditions.

### Response of $g_{m}$ and reassimilation to $C_{a}$ and $I_{\mathrm{inc}}$

We used the reaction‐diffusion model to calculate the apparent $g_{m}$ for each leaf type and each scenario of (photo)respired CO_2_ release. We first used the model to calculate $A_{N}$, $C_{i}$ and $C_{c}$ as described by Berghuijs *et al*. (2017). Next, we re‐arranged Eqn (Eqn 2) to $g_{m}=A_{N}/\left(C_{i}-C_{c}\right)$ to calculate $g_{m}$. We calculated both $g_{m}$ and the fraction of (photo)respired CO_2_ that is reassimilated, $f_{\mathrm{reass}}$, as described by Berghuijs *et al*. (2017), for various levels of $C_{a}$, O and $I_{\mathrm{inc}}$.

### Method to identify most likely locations of (photo)respired CO_2_ release

We calculated the Akaike's Information Criterion (AIC) (Akaike, 1974) for each combination of measured and simulated response curves, for each leaf type and for each scenario. For details, see Methods S1.

## Results

### Estimation of *R*_d_

**Figure 1:**
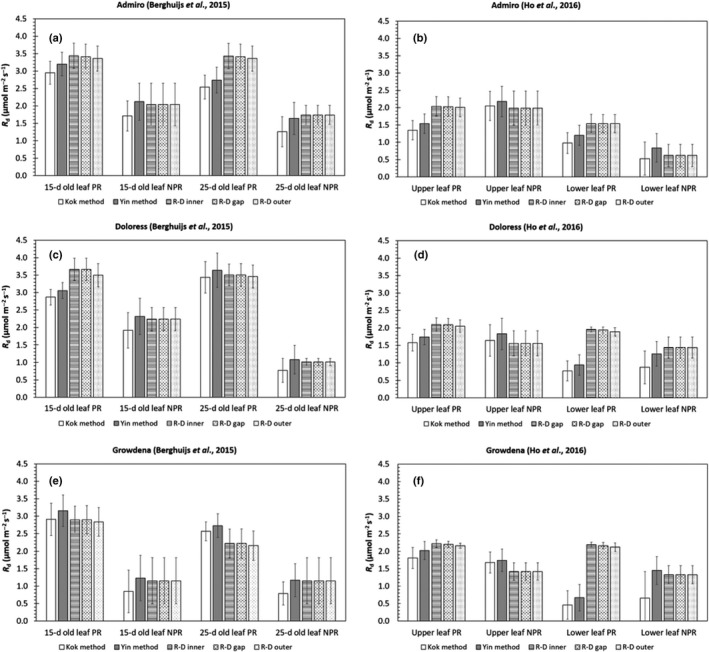
Estimates of day respiration, $R_{d}$, either estimated by the Kok (1948) method, the Yin *et al*. (2009) method or by the reaction‐diffusion model (R‐D). Data collected by Berghuijs *et al*. (2015) (a, c, e) and Ho *et al*. (2016) (b, d, f) for three cultivars (a,b: Admiro, c,d: Doloress, e,f: Growdena) and two leaf ages (either 15‐ and 25‐d‐old leaves or upper leaves and lower leaves) were used for estimation. Estimates were made both for photorespiratory (PR) and nonphotorespiratory (NPR) conditions. *R*_d_ was estimated by the reaction‐diffusion for three diffferent scenarios: (photo)respired CO_2_ was released either in the inner cytosol (RD‐inner), the cytosol gaps (RD‐gaps) or the outer cytosol (RD‐outer). In each panel, the length of the error represents one standard deviation.

We used the reaction‐diffusion model to estimate $R_{d}$ for the leaf types in the datasets of Berghuijs *et al*. (2015) (Fig. 1a,b; Table S1) and Ho *et al*. (2016) (Fig. 1c,d; Table S2). Additionally, we estimated $R_{d}$ by linear regression for the Yin and Kok methods. In 11 of 12 cases, the $R_{d}$ values estimated by the reaction‐diffusion model under photorespiratory conditions were higher than the $R_{d}$ values under nonphotorespiratory conditions.

The values of $R_{d}$ estimated by the reaction‐diffusion model did not differ much for the different assumed positions of (photo)respired CO_2_ release. In all instances, the values of $R_{d}$ estimated by the Yin method were higher than the $R_{d}$ values estimated by the Kok method. In all cases, the values of $R_{d}$ estimated by the Yin method under nonphotorespiratory conditions were close to the values estimated by the reaction‐diffusion model (Fig. 1b,d). Under photorespiratory conditions, this was not always the case (Fig. 1a,c). The values of $R_{d}$ estimated by all of the methods did not differ consistently between leaf ages or leaf types.

### Determination of $T_{p}$ and $V_{\mathrm{cmax}}$

Because of the similar estimates for $R_{d}$, there were also almost no differences for the estimates of $T_{p}$ for the same leaf types among different assumed locations of (photo)respired CO_2_ release (Table S3). The estimate of $V_{\mathrm{cmax}}$ for each leaf type was lower if the (photo)respired CO_2_ release was assumed to take place in the inner cytosol than if it was to take place in the cytosol gaps (Table S3). When (photo)respired CO_2_ release took place in the outer cytosol, the estimate of $V_{\mathrm{cmax}}$ was always of the same order of magnitude as its standard error.

### Model validation

Figs S1 and S2 show a comparison between measured and simulated CO_2_ and light response curves, respectively, for each scenario of the location (photo)respired CO_2_ release. They display only the part of the curves for which the measured data were not used for parameterization. Under most conditions, there was a good agreement between the measured and simulated net CO_2_ assimilation rate for any scenario. However, under photorespiratory conditions the model that assumes (photo)respired CO_2_ release in the outer cytosol tended to underestimate the net CO_2_ assimilation rate more than the other two scenarios, under low CO_2_ concentrations in the CO_2_ response curves and high irradiances in the light response curves.

### Response of $g_{m}$, and reassimilation to $C_{a}$ and *I*_inc_

**Figure 2:**
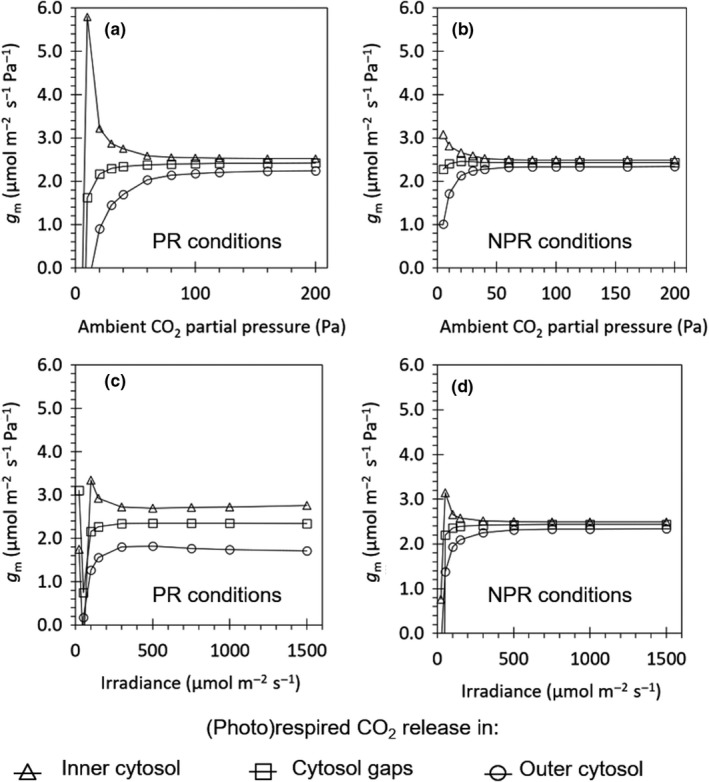
Response of the simulated apparent mesophyll conductance ($g_{m}$) to increased ambient CO_2_ concentrations (a,b) or light intensities (c,d) under ambient oxygen (O=21kPa) concentrations (a, c) and low oxygen (O=2kPa) concentrations (b, d). The CO_2_ response curves were measured under saturating light (*I*_inc_ = 1500 μmol m^−2^ s^−1^) in 15‐d‐old Admiro leaves from the Berghuijs *et al*. (2015) dataset. The light response curves at low oxygen concentrations were simulated under high CO_2_ concentrations (*C*_a_ **= 40 Pa). Light response curves at high oxygen concentrations were measured at ambient CO_2_ concentrations (*C*_a_ = 40 Pa). The release of (photo)respiratory CO_2_ is assumed to take place either in the inner cytosol (triangles), the cytosol gaps (squares) or the outer cytosol (circles). The lines connect each of the triangles, squares or circles.

Figure 2 shows how $g_{m}$ responded to $C_{a}$ and to $I_{\mathrm{inc}}$ for the case of 15‐d‐old leaves of cv Admiro. The relationship for other leaf types showed a similar trend, and is therefore not shown here. If (photo)respired CO_2_ release took place in the outer cytosol or in the cytosol gap, $g_{m}$ increased with increasing $C_{a}$. If (photo)respired CO_2_ release took place in the inner cytosol, $g_{m}$decreased with $C_{a}$. $g_{m}$ was always larger if (photo)respiratory CO_2_ release took place in the inner cytosol than in the cytosol gaps, and in the cytosol gaps than in the outer cytosol. For each scenario, $g_{m}$ tended to approach an equilibrium value at a high *C*_a_, and this equilibrium value was the same for 21 and 2 kPa O_2_ conditions for the same leaf type. See Methods S2 for further comments.

**Figure 3:**
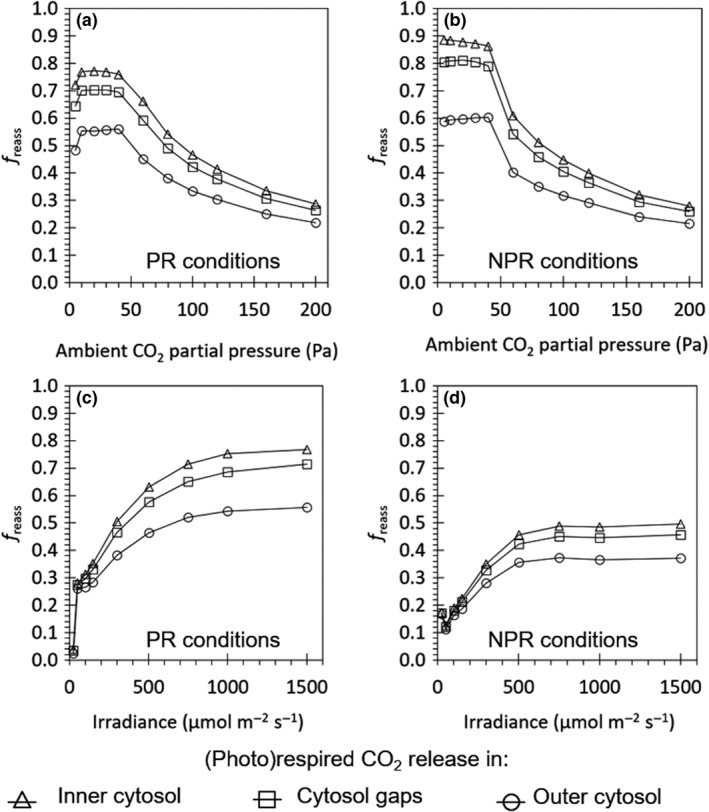
Response of the simulated fraction of reassimilation (photo)respired CO_2_ (*f*_reass_) to increased ambient CO_2_ concentrations (a,b) or light intensities (c,d) under ambient oxygen (O=21kPa) concentrations (a, c) and low oxygen (O=2kPa) concentrations (b, d). The CO_2_ response curves were measured under saturating light (*I*_inc_ = 1500 μmol m^−2^ s^−1^) in 15‐d‐old Admiro leaves from the Berghuijs *et al*. (2015) dataset. The light response curves at low oxygen concentrations were simulated under high CO_2_ concentrations (*C*_a_ **= 40 Pa). Light response curves at high oxygen concentrations were measured at ambient CO_2_ concentrations (*C*_a_ = 40 Pa). The release of (photo)respiratory CO_2_ is assumed to either take place in the inner cytosol (triangles), the cytosol gaps (squares) or the outer cytosol (circles). The lines connect each of the triangles, squares or circles.

Figure 3 shows the response curves of $f_{\mathrm{reass}}$ to $C_{a}$ and to $I_{\mathrm{inc}}$, for 15‐d‐old Admiro leaves from Berghuijs *et al*. (2015), and this relationship are similar for the other leaf types. The relationship with $C_{a}$ was sigmoidal, and that with $I_{\mathrm{inc}}$ was in a saturation shape, under both oxygen concentrations.

### Simulations under conditions of the Laisk method

**Figure 4:**
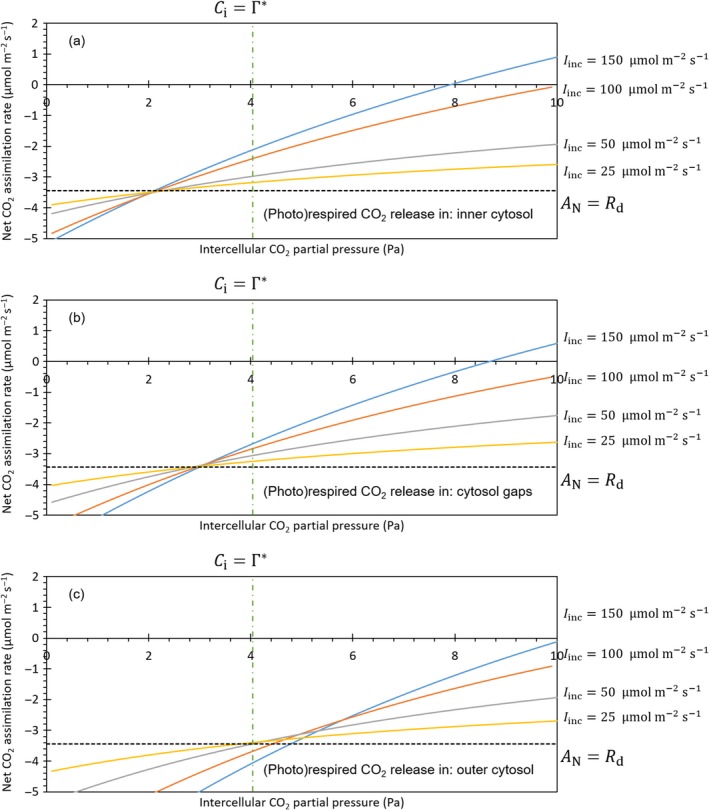
Simulated response curve of the net CO_2_ assimilation rate (*A*_N_) to intercellular CO_2_ partial pressures (*C*_i_) under the conditions of the Laisk method for different scenarios of (photo)respired CO_2_ release. The solid lines represent CO_2_ response curves simulated at different irradiances *I*_inc_ (150, 100, 50 and 25 μmol m^−2^ s^−1^). The dashed line represents the net CO_2_ assimilation rate which equals the negative input value of the day respiration rate (*R*_d_). The dashed‐dotted line represents the intercellular partial pressure which equals the CO_2_ compensation point (Γ*). (Photo)respired CO_2_ is released either in the inner cytosol (a), the cytosol gaps (b) or the outer cytosol (c).

We simulated $A_{N}-C_{i}$ curves under different irradiances, for 15‐d‐old Admiro leaves (Fig. 4) using the $R_{d}$ values that were previously estimated for each scenario of (photo)respired CO_2_ release (Figs S1, S2). If (photo)respired CO_2_ release took place in the inner cytosol, the curves had about the same intersection point at $C_{i}=2.1,\mathrm{Pa}$. If (photo)respired CO_2_ release took place in the cytosol gaps, the curves had about the same intersection point in $C_{i}=3.0,\mathrm{Pa}$. For both scenarios, the common intersection points of the curves also were intersected with the line $A_{N}=-R_{d}$. If (photo)respired CO_2_ release took place in the outer cytosol, the curves did not intersect at the same value of $C_{i}$. Instead, individual curves intersected in intercellular CO_2_ partial pressures of 4.3, 4.7, 5.1, 5.4 and 5.8 Pa, respectively. The values of $A_{N}$ in these intersection points were higher than $-R_{d}$. The intersection points were not obtained by the line $C_{i}=\Gamma^{*}$ in any of the three scenarios.

### Likely locations of (photo)respired CO_2_

We calculated ΔAIC for each combination of leaf type and scenario for each measured response curve type (Tables S4, S5). The ΔAIC values are bold if ΔAIC≤2, indicating the corresponding scenario has substantial support (Burnham & Anderson, 2004). There was only one case (Admiro lower leaf CO_2_ response curves at ambient O_2_; Table S5) in which the scenario that assumed (photo)respired CO_2_ release in the outer cytosol had substantially greater support than the scenario that assumed the release in the inner cytosol. There also was only one case in which the scenario that assumed (photo)respired CO_2_ release in the cytosol gaps had more support than the other two scenarios. In the other 46 out of 48 cases, the model in which (photo)respired CO_2_ release took place in the inner cytosol had either the most support or substantial support relative to the best model. In all cases, all three scenarios had substantial support for the light response curves under nonphotorespiratory conditions.

## Discussion

### Use of a simple reaction‐diffusion model as a tool to assess $R_{d}$

Reaction‐diffusion models have been used as powerful tools to investigate mesophyll conductance (*g*_m_) and its response to various environmental and physiological factors (Tholen & Zhu, 2011; Ho *et al*., 2016). Here, we explored using the simple reaction‐diffusion model of Berghuijs *et al*. (2017) to investigate the day respiration rate (*R*_d_). Reaction‐diffusion models certainly have limitations. The most relevant one in the context of this study is that these models require prefixed diffusion coefficients as input, whose values are hard to measure. Therefore, we had to adopt these from previous studies (Gutknecht *et al*., 1977; Evans *et al*., 2009; Fanta *et al*., 2012; Ho *et al*., 2016). Additionally, we simplified the leaf structure to a single rectangular cuboid chloroplast, surrounded by a cytosol layer (Berghuijs *et al*., 2017). This simplification simulates the leaf tissue as a 2D computational domain, while assuming that the third dimension is homogeneous. These simplifications can potentially affect the simulated results. However, Berghuijs *et al*. (2017) validated the simple model by comparing the results with those generated by a complex 3D model (Ho *et al*., 2016). Here, we further validated the model by comparing measured (Berghuijs *et al*., 2015; Ho *et al*., 2016) and simulated net CO_2_ assimilation rates (Figs S1, S2). Our model had various advantages. First, computational time was greatly reduced, which made it feasible to use the model directly to estimate $R_{d}$. Second it can be parameterized using a limited number of leaf anatomical parameters. We showed that the model, when combined with anatomical parameters, gas exchange and chlorophyll fluorescence data, adds to the literature by providing an additional method to indirectly estimate *R*_d_ under either photorespiratory or nonphotorespiratory conditions. We chose leaf anatomical properties that have been measured in combination with gas exchange in various previous studies (Syvertsen *et al*., 1995; Tosens *et al*., 2012; Galmes *et al*., 2013; Retta *et al*., 2016b; Ouyang *et al*., 2017).

### Estimation of *R*_d_ by the Kok method and the Yin method

Reassimilation and other processes can compromise the indirect estimation of *R*_d_ based on gas exchange data. We first examined estimates for nonphotorespiratory conditions where reassimilation is not relevant because then CO_2_ released by (photo)respiration does not contribute much to increasing ribulose biphosphate (RuBP) carboxylation (Busch *et al*., 2013). In most cases, $R_{d}$values estimated by the Kok method were smaller than estimates by the reaction‐diffusion model, although these differences are sometimes small as the standard deviations overlap. The estimates of $R_{d}$ obtained from the reaction diffusion model and the ones from the Yin method under nonphotorespiratory conditions are very similar (Figs 1, 2). $R_{d}$estimates by the Kok method under photorespiratory conditions also were smaller than the estimates by the Yin method (Fig. 1), in line with Yin *et al*. (2011). Our reaction‐diffusion model, like the Yin method, considers the decrease of $\Phi_{2}$ with increasing irradiance (Genty & Harbinson, 1996), which occurs even within the low‐irradiance range (Yin *et al*., 2009, 2011). The Kok method underestimates *R*_d_ because it neglects this dependence of $\Phi_{2}$ on irradiance. Recognizing the decrease of $\Phi_{2}$ with increasing irradiance also avoids the underestimation of the quantum yield of CO_2_‐assimilation (Yin *et al*., 2014).

For photorespiratory conditions, estimates of *R*_d_ by the reaction‐diffusion model were higher than those by the Yin method, let alone by the Kok method, for most leaf types (Fig. 1). The underestimation of *R*_d_ by the Yin method relative to the *R*_d_ estimated by the reaction‐diffusion was 10.5–13.0%, depending on the scenario that the reaction‐diffusion model assumed with regard to the location of (photo)respired CO_2_ release (Fig. S3). Under photorespiratory conditions, chloroplast CO_2_ concentration (*C*_c_) decreases significantly with increased light under low light intensities (Farquhar & Busch, 2017). Such variation was generated using the FvCB model coupled with the *g*_m_ model of Eqn (Eqn 2), which has a similar form as a stomatal conductance (*g*_s_) model. Therefore, the generated variation of *C*_c_ with increasing irradiance is similar to the measured pattern for the decrease of intercellular CO_2_ concentration (*C*_i_) with light intensity (Berghuijs *et al*., 2015). The decrease of *C*_c_ with irradiance is a common result when *g*_m_ is finite and is greatest when *g*_m_ is smallest (Farquhar & Busch, 2017). Similarly, our reaction‐diffusion framework, explicitly modelling CO_2_ sources, diffusion and sinks, accounts for the variation of *C*_c_ with increasing irradiance. By contrast, the linear regression procedure of the Kok method or the Yin method implicitly assumes that *C*_c_ does not vary with irradiance, when applied under photorespiratory conditions. Ignoring this variation of *C*_i_ or *C*_c_ has been shown by Kirschbaum & Farquhar (1987) and Farquhar & Busch (2017) to lead to an underestimation of *R*_d_ (see also Buckley *et al*., 2017). Therefore, we conclude that the Yin method underestimates *R*_d_ for photorespiratory conditions by neglecting the variation of *C*_c_ with increasing irradiance.

To what extent is this variation of *C*_c_ with increasing irradiance associated with the reassimilation by (photo)respired CO_2_? For nonphotorespiratory conditions that are achieved with a very low O_2_ concentration, both a *g*_m_ model and the reaction‐diffusion model can predict a decline of *C*_c_ with increasing irradiance (results not shown), but with a negligible effect on leaf photosynthesis. By contrast, for photorespiratory conditions, this decline is highly relevant as it affects the rate of RuBP carboxylation. The FvCB model, when combined with *g*_s_ and *g*_m_, accounts for reassimilation of (photo)respired CO_2_ (Tholen *et al*., 2012; Von Caemmerer, 2013; Yin & Struik, 2017). A high *g*_m_ value could predict little drawdown of *C*_c_ from *C*_i_ with increasing irradiance and a low intracelluar reassimilation. Therefore, for photorespiratory conditions, the modelled variation of *C*_c_ with irradiance indirectly reflects the contribution of (photo)respired CO_2_ release to *C*_c_, therefore, to reassimilation. This assertion is supported by the similarity between the above‐stated percentages of *R*_d_ underestimation by the Yin method (10.5–13.0%) and the values of *f*_reass_ we estimated for the low‐irradiance range (Fig. 3). The small difference in *f*_reass_ at low light among the three possible positions of mitochondria (Fig. 3) is also in line with the small difference among *R*_d_ estimates in different scenarios (Fig. 1). Although the placement of mitochondria is known to affect *f*_reass_ (Yin & Struik, 2017), our reaction‐diffusion model predicts that such an effect of the scenario is most expressed under high‐light conditions (Fig. 3).

Because of the above differences in handling the irradiance‐dependence of *C*_c_ and reassimilation, the relative value of *R*_d_ estimated for the photorespiratory vs nonphotorespiratory conditions by the Yin method and the reaction‐diffusion model differed. The estimates of $R_{d}$ by the Yin method were either higher or lower in one than in the other conditions, whereas those by the reaction‐diffusion model were always lower for nonphotorespiratory than for photorespiratory conditions (Fig. 1). This is in agreement with results from Buckley *et al*. (2017), who showed that *R*_dk_ was higher at 21% than at 2% O_2_ in developing leaves of *Vicia faba*. Respiration is a process where O_2_ is the substrate (Tcherkez *et al*., 2017b) and respiratory rates measured in terms of O_2_ and CO_2_ exchange may not be equal (Gauthier *et al*., 2018). However, the amount of respiratory CO_2_ release at the low O_2_ concentration, as applied for measurements under nonphotorespiratory conditions, will likely decrease relative to that under ambient O_2_ conditions. The Yin method is theoretically valid for nonphotorespiratory conditions only (Yin *et al*., 2011). However, it is previously unknown to what extent *R*_d_ estimated from nonphotorespiratory conditions can be used for photorespiratory conditions. Based on our results and those found in literature (Buckley *et al*., 2017), we conclude that the *R*_d_ estimate obtained under nonphotorespiratory conditions by the Yin method cannot be used as a replacement for *R*_d_ under photorespiratory conditions.

This conclusion also applies to the Kok method. One assumption when using the Kok method or the Yin method under photorespiratory conditions is that *C*_c_ is constant for different light intensities. However, practically, it is impossible to design an experiment where *C*_c_ is maintained constant across various irradiances because *g*_s_, *g*_m_ and *A*_N_ are not known beforehand (Buckley *et al*., 2017). Our analysis shows the power of using reaction‐diffusion models parameterized with standard diffusion coefficients (Berghuijs *et al*., 2017) and leaf anatomical measurements (Berghuijs *et al*., 2015) to estimate *R*_d_, which can account for the decrease of both $\Phi_{2}$ and *C*_c_ with increasing irradiance.

### Estimation of *R*_d_ by the Laisk method

The Laisk method relies on measurements at low *C*_i_, the conditions having high photorespiration, but it theoretically has problems if *r*_m_ is significant, especially under the framework of multiple components of *r*_m_ (Tholen *et al*., 2012). Yin *et al*. (2011) stated that *R*_d_ estimated by the Laisk method was comparable with the estimates by the Yin method for photorespiratory conditions. Gong *et al*. (2018) showed that the Laisk method underestimates *R*_d_ when compared with their isotopic disequilibrium method that directly estimates *R*_d_.

The results of our simulations of CO_2_ response curves under the conditions of the application of the Laisk method (Fig. 4) actually show that assumptions regarding the location of (photo)respired CO_2_ release affect the estimates it obtains. If (photo)respired CO_2_ is assumed to be released in the outer cytosol, the curves do not intersect in a single point. Moreover, each of the intersection points between two curves has a higher net CO_2_ assimilation rate than the prefixed –*R*_d_. Based on their model, which implicitly assumes (photo)respired CO_2_ release in the outer cytosol, Tholen *et al*. (2012) also indicates that the Laisk method will underestimate *R*_d_. Our simulations show that in the other two scenarios, CO_2_ response curves actually do intersect in $A_{N}=-R_{d}$. This shows that if one of these two scenarios is true, Laisk's method yields a good estimate of $R_{d}$.

In an application of the Laisk plot, it is still required that all CO_2_ response curves share a single intersection point. This issue can be solved by the fitting procedure as described by Yin *et al*. (2011) for the Laisk method or the slope‐intercept regression analysis as applied by Walker & Ort (2015). However, the Laisk linear plot to estimate *R*_d_ should be made as a function of *C*_c_; for that $g_{m}$ needs to be known. A dilemma is that *g*_m_ can be estimated only after *R*_d_ is known (Harley *et al*., 1992). Again, the reaction‐diffusion model does not have this problem as it does not require $g_{m}$ as an input beforehand. Nevertheless, relying on *g*_m_ values indirectly derived from an established relationship between *g*_m_ and *g*_s_, Gong *et al*. (2018) showed that *R*_d_ estimated by the Laisk method does not depend on whether it is based on *C*_i_ or *C*_c_.

### Estimates of photosynthetic parameters and mesophyll conductance in relation to the position of mitochondria

The estimate of $V_{\mathrm{cmax}}$ was always higher if (photo)respiratory CO_2_ release took place in the cytosol gap than in the inner cytosol (Table S3). Because the reassimilation of (photo)respiratory CO_2_ was higher if (photo)respiratory CO_2_ was released in the inner cytosol than in the cytosol gaps (Fig. 3), the model compensated for the lower reassimilation by a higher RuBP carboxylation under Rubisco limited conditions, thereby resulting in a higher estimated $V_{\mathrm{cmax}}$. If (photo)respiratory CO_2_ was released in the outer cytosol, the standard error was very high (Table S3), possibly because the model cannot fully compensate for the discrepancy between its prediction of $A_{N}$ and the measured $A_{N}$ for this scenario by estimating a high value for $V_{\mathrm{cmax}}$.

For all leaf types, our reaction‐diffusion model generated the same trend in the response of $g_{m}$ to different values of $C_{a}$ and $I_{\mathrm{inc}}$ (Fig. 2). If (photo)respired CO_2_ release was assumed to take place in the inner cytosol, $g_{m}$ decreased with an increase in $C_{a}$. The shape of this response was similar to the response of $g_{m}$ to $C_{i}$ (when $C_{i}$ was above certain values) reported in various studies (Flexas *et al*., 2007; Yin *et al*., 2009; Tholen & Zhu, 2011). The apparent *g*_m_ model as used in these studies was Eqn (Eqn 2). This model assumes that the mitochondria are located closely behind the chloroplasts as if that (photo)respired CO_2_ were released in the same compartment as RuBP carboxylation does (Tholen & Zhu, 2011; Yin & Struik, 2017). If (photo)respired CO_2_ was to release in the outer cytosol or in the cytosol gaps, the shape of the response was more similar to the one calculated by Tholen *et al*. (2012) using a resistance model based on the same assumption. Xiao & Zhu (2017) also found similar differences in the shape of the response curve of $g_{m}$ to $C_{i}$ depending on the position of the mitochondria relative to the chloroplasts.

### The most likely position of mitochondria relative to the chloroplasts

In a vast majority of cases, the scenario for (photo)respired CO_2_ release in the outer cytosol had less support than the scenario that assumed (photo)respired CO_2_ release in the inner cytosol (Tables S4 and S5). The consequences of this finding is that, at least in tomato, two‐resistance models (Tholen *et al*., 2012; Berghuijs *et al*., 2015) that implicitly assume (photo)respired CO_2_ release in the outer cytosol are less likely than the classical single mesophyll resistance models.

This assertion agrees generally with experimental observations like (Hatakeyama & Ueno, 2016), who reported that for 10 C_3_ grasses, on average 80% of the mitochondria are located closely on the vacuole side of chloroplasts in mesophyll cells. Sage & Sage (2009) and Busch *et al*. (2013) had a similar observation for rice and wheat, who even indicated that chloroplast covers > 95% of the mesophyll periphery with a high *S*_c_ : *S*_m_ ratio that provides an effective mechanism to trap and re‐assimilate (photo)respired CO_2_. The classical resistance model, Eqn (Eqn 2), works best if mitochondria are exclusively located closely behind chloroplasts and the *S*_c_ : *S*_m_ ratio is very close to 1.0, whereas the two‐resistance model of Tholen *et al*. (2012) works if mitochondria are located predominantly in the outer cytosol combined with a low *S*_c_ : *S*_m_ and little cytosol resistance (Yin & Struik, 2017). Although the reality may be somewhere between these two extremes, our analysis in Tables S4 and S5 suggests that the classical *g*_m_ model, Eqn (Eqn 2), is closer to reality in the two experiments for tomato. It should be noted that the *S*_*c*_ : S_m_ measurements that were used in this study (between 0.84 and 0.96) (Berghuijs *et al*., 2015) were all at the higher end of the values mentioned in literature for various species. For instance, considerably lower ranges have been reported for *Arabidopsis thaliana* (0.43‐0.75) (Tholen *et al*., 2008). As *S*_c_ : *S*_m_ decreases with leaf aging and varies with species (Busch *et al*., 2013) and with environment (Ouyang *et al*., 2017), it may be hard to ascertain which model of the two is closer to reality.

## Concluding remarks

Our reaction‐diffusion model can estimate *R*_d_ without making implicit assumptions regarding reassimilation, position of mitochondria, mesophyll conductance and the variability of *C*_c_ at low light conditions. In these aspects, our model provides a better tool to estimate *R*_d_ than the Laisk, Kok and Yin methods. However, if there are no leaf anatomical data available, it depends on the available data which of the existing models has to be applied. Table 2 shows an overview of the advantages and disadvantages of each method. With leaf anatomical data available, reaction‐diffusion models have previously shown to be useful to study the mechanisms of mesophyll conductance and reassimilation (Tholen & Zhu, 2011; Ho *et al*., 2016; Retta *et al*., 2016a, 2017; Berghuijs *et al*., 2017). We demonstrate here that they also can estimate *R*_d_ and photosynthetic parameters. We recommend further research to collect datasets containing leaf anatomical parameters in combination with gas exchange and chlorophyll fluorescence measurements to make the best possible use of our method.

**Table 2:** Overview of advantages and disadvantages of estimation methods for *R*_d_

Method	Advantages	Disadvantages
Kok method	Does not require chlorophyll fluorescence measurements	Does not consider the increase of *C*_c_ with decreased irradiance
	Does not require leaf anatomical measurements	Is theoretically only valid under nonphotorespiratory conditions
		Does not account for the decrease of Φ_2_ with increased irradiance
Yin method	Does not require leaf anatomical measurements	Requires chlorophyll fluorescence measurements
	Accounts for the decrease of Φ_2_ with increased irradiance	Does not consider the increase of *C*_c_ with decreased irradiance
		Is theoretically only valid under nonphotorespiratory conditions
Laisk method	Does not require chlorophyll fluorescence measurements	Requires an assumption of no mesophyll resistance
	Does not require leaf anatomical measurements	Estimate is affected by the position of mitochondria relative to the chloroplasts
	Partly considers the reassimilation of photorespired CO_2_	Is applied at very low CO_2_ concentrations and requires gas leakage corrections
	Applies to photorespiratory conditions	
Reaction diffusion model	Does not require an estimate of mesophyll conductance	Requires chlorophyll fluorescence measurements
	Accounts for the decrease of Φ_2_ with increased radiation	Requires leaf anatomical parameters to parameterize the geometry
	The placement of mitochondria relative to the chloroplasts can be defined explicitly	Requires CO_2_ diffusion coefficients for different mesophyll compartments
	Is theoretically valid under photorespiratory conditions	Has to be solved numerically

## Author contributions

HNCB planned the research and wrote the first draft of the manuscript; HNCB and XY ran the simulations; HNCB, XY, QTH, MAR, BMN and PCS interpreted the simulations; and HNCB, XY, QTH, MAR, BMN and PCS wrote the final manuscript.

## Supporting information

Please note: Wiley Blackwell are not responsible for the content or functionality of any Supporting Information supplied by the authors. Any queries (other than missing material) should be directed to the *New Phytologist* Central Office.

**Fig. S1** Measured vs simulated CO_2_ response curves under photorespiratory and nonphotorespiratory conditions.
**Fig. S2** Measured vs simulated light response curves under photorespiratory and nonphotorespiratory conditions.
**Fig. S3** Day respiration rate estimates obtained by the reaction diffusion model vs estimates obtained by the Yin method.
**Fig. S4** Schematic overview of the flow of the program that was used to estimate parameter values with the reaction diffusion model.
**Methods S1** Determination of Akaike's Information Criterion.
**Methods S2** Comments on *g*_m._
**Notes S1** Source code to estimate *R*_d_ and *V*_cmax_.
**Notes S2** Code of M files.
**Table S1** Estimates of the lumped calibration factors and the day respiration rates obtained by various methods using data from Berghuijs *et al*. (2015).
**Table S2** Estimates of the lumped calibration factors and the day respiration rates obtained by various methods using data from Ho *et al*. (2016).
**Table S3** Estimates of the maximum RuBP carboxylation rate by Rubisco and the triose phosphate utilization rates obtained by the reaction‐diffusion model for different scenarios of (photo)respired CO_2_ release.
**Table S4** Akaike's information criteria for different combinations of leaf age, cultivar, photorespiratory conditions and scenarios for the release of (photo)respired CO_2_ using the reaction‐diffusion model and data from Berghuijs *et al*. (2015).
**Table S5** Akaike's information criteria for different combinations of leaf age, cultivar, photorespiratory conditions, and scenarios for the release of (photo)respired CO_2_ using the reaction‐diffusion model and data from Ho *et al*. (2016).
