# Supplementary material for: Using a reaction‐diffusion model to estimate day respiration and reassimilation of (photo)respired CO 2 in leaves
Source: New Phytol. 2019 May 11;223(2):619–31. doi: 10.1111/nph.15857 (PMC6618012; doi:10.1111/nph.15857)
Supplement: Supplementary file 1 — Fig. S1 Measured vs simulated CO2 response curves under photorespiratory and nonphotorespiratory conditions. Fig. S2 Measured vs simulated light response curves under photorespiratory and nonphotorespiratory conditions. Fig. S3 Day respiration rate estimates obtained by the reaction diffusion model vs estimates obtained by the Yin method. Fig. S4 Schematic overview of the flow of the program that was used to estimate parameter values with the reaction diffusion model. Methods S1 Determination of Akaike's Information Criterion. Methods S2 Comments on g m. Notes S1 Source code to estimate R d and V cmax. Notes S2 Code of M files. Table S1 Estimates of the lumped calibration factors and the day respiration rates obtained by various methods using data from Berghuijs et al. (2015). Table S2 Estimates of the lumped calibration factors and the day respiration rates obtained by various methods using data from Ho et al. (2016). Table S3 Estimates of the maximum RuBP carboxylation rate by Rubisco and the triose phosphate utilization rates obtained by the reaction‐diffusion model for different scenarios of (photo)respired CO2 release. Table S4 Akaike's information criteria for different combinations of leaf age, cultivar, photorespiratory conditions and scenarios for the release of (photo)respired CO2 using the reaction‐diffusion model and data from Berghuijs et al. (2015). Table S5 Akaike's information criteria for different combinations of leaf age, cultivar, photorespiratory conditions, and scenarios for the release of (photo)respired CO2 using the reaction‐diffusion model and data from Ho et al. (2016). [file NPH-223-619-s001.pdf]

New Phytologist Supporting Information

Article title:

Using a reaction-diffusion model to estimate day respiration and re-assimilation of (photo)respired CO_2 in leaves

Authors:

Herman N.C. Berghuijs, Xinyou Yin, Q. Tri Ho, Moges A. Retta, Bart M. Nicolaï, Paul C. Struik

Acceptance date: April 5 2019

The following Supporting Information is available for this article:

Methods S1: Determination of Akaike's Information Criterion

In order to calculate Akaike's Information Criterion, we first minimized the negative log likelihood L for the standard deviation σ for each curve type and cultivar separately:

$$L_{\min} = \frac{N}{2} \cdot \ln(2 \cdot \pi) + \frac{N}{2} \cdot \log(\sigma^2) + \frac{1}{2\sigma^2} \cdot \sum_{i=1}^{N} \left(A_{\mathrm{N},i} - \overline{A_{\mathrm{N},i}}\right)^2 \qquad \text{(S1.1)}$$

$$\mathrm{AIC} = 2 \cdot L_{\min} + 2 \cdot k \qquad \text{(S1.2)}$$

where $L_{\min}$ is the minimum negative log likelihood assuming normally distributed residuals. k is the number of estimated parameters. In this study, the different models that we compare have the same number of estimated parameters; only the location of (photo)respiratory CO_2 release was different among the different models. Therefore, the term $2 \cdot k$ is the same for any of the scenarios and can be omitted from equation (6). $A_{\mathrm{N,i}}$ is the measured A_{N} for a certain set of environmental conditions. $\overline{A_{\mathrm{N,\imath}}}$ is the simulated A_{N} for these conditions. N is the total number of measurements for this curve type for this cultivar. For each scenario, curve type and cultivar, we calculated ΔAIC as:

$$\Delta\mathrm{AIC_i} = \mathrm{AIC_i} - \mathrm{AIC_{min}} \qquad \text{(S1.3)}$$

where $\mathrm{AIC_{min}}$ is the lowest AIC value for each scenario of (photo)respired CO_2 release (in the inner cytosol, cytosol gaps or outer cytosol). The model, for which $\Delta\mathrm{AIC} = 0$, is considered the best model.

According to Burnham and Anderson (2004), ΔAIC represents the information loss if an alternative model is fitted to the data, rather than the best model. They stated that the alternative model has "substantial support" if $\Delta\mathrm{AIC} \leq 2$. We adopted this interpretation of ΔAIC in our study.

Methods S2: Comments on g_m

If C_a is very low (5 Pa or less), g_m turns negative. Tholen and Zhu (2011) also observed this phenomenon. They explained this by the fact that under very low CO_2 levels, (photo)respired CO_2 release in the cytosol is very high relative to RuBP carboxylation in the chloroplast. Under these conditions, a substantial amount of (photo)respired CO_2 leaks out of the mesophyll cell, which can result in an increase in C_i. This can result in a situation, where $C_i - C_c$ is positive, while A_N is still negative. As the apparent mesophyll conductance is defined as $g_m = \frac{A_N}{C_i - C_c}$, this situation can lead to a negative g_m.

Notes S1: Source code to estimate $\boldsymbol{R_\mathrm{d}}$ and $\boldsymbol{V_\mathrm{cmax}}$

Notes S1.1 Introduction

We developed a code to use a reaction diffusion model to estimate the FvCB model parameters R_d (rate of day respiration; μmol CO_2 m^{-2} s^{-1}) and V_cmax (maximum rate of RuBP carboxylation by Rubisco; μmol CO_2 m^{-2} s^{-1}) from leaf anatomical measurements and simultaneously measured gas exchange and chlorophyll fluorescence data. We estimated these parameters for three different scenarios; CO_2 produced by (photo)respiration was either released in 1) the inner cytosol, 2) the outer cytosol, or 3) the cytosol gaps. Although the model source code has already been published by Berghuijs *et al.* (2017), this publication did not include the code that was used to estimate R_d and V_cmax. Therefore, we provide this code here for users who are interested in our approach. The aim of this document is to explain how this code works.

Notes S1.2 Requirements

The model was built using the commercial Finite Element Methods software package COMSOL Multiphysics version 5.1 (COMSOL AB, Sweden). We used the COSMOL with MATLAB LiveLink extension (COMSOL AB, Sweden) to convert the COMSOL model into a MATLAB code. In order to run the code, both COMSOL 5.1 (or a newer version) and MATLAB 2014b (or a newer version) have to be available on the computer.

Notes S1.3 Overview of M-files

The code consists of a number of M-files. These are:

- main.m
- buildModelGaps.m
- buildModelInner.m
- buildModelOuter.m
- calculateResidualRd.m
- calculateResidualVcmax.m
- dataRd.m
- dataVcmax.m
- estimateRd.m
- estimateVcmax.m
- initiateModel.m

- initiateSolver.m
- parameter.m
- setParameters.m
- solveModel.m

Notes S1.4 Running the estimation procedure

In order to run the code, all files need to be stored in one single work directory. This work directory is specified in main.m at line 48:

```
currentDirectory = 'M:\My Documents\Workdirectory';
```

If a user wants to run the code, but the M-files are stored in another directory, the value of the variable `currentDirectory` needs to be adjusted. The main function of the program is main.m. If this M-file is run, the program estimates V_{cmax} and R_d from gas exchange data for the three different scenarios. The flow chart of the program to estimate V_{cmax} and R_d for the scenario that assumes (photo)respiratory CO_2 release in the inner cytosol is shown schematically in Figure ~~SX.1~~. The flows to estimate these parameters for the other two programs are very similar. The only difference is that the file buildModelInner.m in the diagram needs to be replaced by BuildModelOuter.m ((photo)respiratory CO_2 release in the outer cytosol) or by buildModelGaps ((photo)respiratory CO_2 release in the cytosol gaps). We will explain the function of each M-file and the M-files that they call in more detail in section 6.

Notes S1.5 User guide

If one runs the source code in Notes S2, parameters are estimated for V_{cmax} and R_d from a sample data set and leaf anatomical properties for three scenarios of the position of mitochondria relative to the chloroplast. In this specific case, the sample data set consists of gas exchange data, chlorophyll fluorescence measurements, and various leaf anatomical properties collected for 15-days old leaves of tomato cultivar Admiro. More details on the collection of these data are described by Berghuijs *et al.* (2015). If one wants to estimate R_d and V_{cmax} from another dataset, a number of variables in the M-files parameters.m, dataRd.m, and dataVcmax.m have to be adjusted. The aim of this section is to describe for each of these files which parameters have to be adjusted and how this can be done.

The M-file parameters.m contains, among others, values for leaf anatomical properties. These variables can be found under the header "Set anatomical parameters":

```
%% Set anatomical parameters
t_CYT=243e-9;        % Cytosol thickness (m);
t_STR=2.5458e-6;     % Stroma thickness (m);
Sm_S=17;             % Ratio of the mesophyll surface area exposed to
                     % the intercellular airspaces to leaf surface area
t_WALL=118e-9;       % Cell wall thickness (m)
Sc_Sm=0.919;         % Ratio of the area of the chloroplast surface facing
                     % the intercellular airspace to the mesophyll surface
                     % area exposed to the intercellular air space.
ratio=2.5;           % Ratio stroma thickness : stroma height
```

Each of these parameters can be adjusted. Additionally, parameters.m contains variables of environmental properties:

```
%% Set environmental parameters
Iinc=1500;          % Irradiance (umol m^-2 s^-1)
Ci=25;               % CO2 partial pressure in intercellular air spaces (Pa)
O=21;                % Oxygen partial pressure (kPa)
```

If the code is used to estimate R_d and V_{cmax}, the variables for irradiance (`Iinc`) and intercellular CO_2 partial pressure (`Ci`) will be overwritten by the M-file setParameters and it is therefore not necessary to replace their values. However, the oxygen partial pressure `O` is not. If one wants to estimate R_d for a dataset measured under non-ambient oxygen levels, for instance non photorespiratory conditions, its value has to be updated. Finally, parameters.m contains a number of physiological variables:

```
%% Set physiological parameters
Vcmax=256;           % Maximum rate of RuBP carboxylation (umol m^-2 s^-1)
ScO=2.6;             % Rubisco specificity (mbar ubar^-1)
Rd=2.46;             % Rate of respiration (umol m^-2 s^-1)
Tp=12.8;             % Rate of triose phosphate utilization (umol m^-2 s^-1)
KmC=26.4;            % Michaelis Menten constant for RuBP carboxylation (Pa)
KmO=16.4;            % Michaelis Menten constant for RuBP oxygenation (kPa);
J=100;               % Linear rate of electron transport (umol m-2 s-1)
```

Since Vcmax and Rd are overwritten during the estimation procedure, there is no need to replace them. Parameter Tp is not overwritten. However, it is not used if the code is used for the sole purpose of estimation, because in these estimations the net CO_2 assimilation rate is either set equal to the electron transport limited net CO_2 assimilation rate (to estimate R_d) or to the Rubisco limited net CO_2 assimilation rate (to estimate V_{cmax}). The remaining parameters are used during the estimation procedure. Those constants can be changed, if necessary.

The M-file dataRd.m consists those vectors that contain data from gas exchange measurements (Berghuijs *et al.*, 2015) and previously calculated linear rates of electron transport (Berghuijs *et al.*, 2017) that are used to estimate R_d:

```
% Observed net CO2 assimilation rate (umol m-2 s-1)
An_dataRd=[5.856395852,2.904632356,-0.299575675,-1.279395273];
%
% Observed ambient CO2 partial pressure (Pa)
Ca_dataRd=[40.04726334,40.02993698,40.03736954,40.0433342];
%
% Observed intercellular CO2 partial pressure (Pa)
Ci_dataRd =[34.4021,36.1239,39.1888,40.4919];
%
% Observed irradiances (umol m-2 s-1)
Iinc_dataRd=[149.3584557,100.3891029,51.48970413,23.60690546];
%
% Calculated rates of electron transport (J = s * PSII), where J is the
% rate of electron transport and s is a lumped calibration faction.
% (umol m-2 s-1)
J_dataRd=[51.96434159,36.29423493,19.29827995,9.031788168];
%
```

The vectors contain mean values for the measured net CO_2 assimilation rate (`An_dataRd`), the ambient CO_2 partial pressure (`Ca_dataRd`), the intercellular CO_2 partial pressure (`Ci_dataRd`), and the irradiance (`Iinc_dataRd`) . It also contains previously calculated rates of linear electron transport. If one wants to estimate R_d from another dataset, each of the variables listed above has to be replaced by values from that new data set.

Similarily, the M-file dataVcmax.m contains vectors with gas exchange data and calculated rates of electron transport as well:

```
%
% Observed ambient CO2 partial pressure (Pa)
Ca_dataVcmax =[29.93387146,20.022583,9.940552521,5.080346298];
%
% Observed intercellular CO2 partial pressure (Pa)
Ci_dataVcmax = [18.98789412,13.17666079,7.961297167,5.39349546];
%
% Observed net CO2 assimilation rate (umol m-2 s-1)
An_dataVcmax = [17.89843384,11.83933345,3.667620745,-0.971040286];
%
% Observed irradiances (umol m-2 s-1)
Iinc_dataVcmax=[1499.87265,1499.819733,1499.69812     1499.603577];
%
% Calculated rates of electron transport (J = s * PSII), where J is the
% rate of electron transport and s is a lumped calibration faction.
% (umol m-2 s-1)
J_dataVcmax=[192.7053082,173.9048405,147.0389258,126.1549297];
%
```

The vectors contain mean values for the measured net CO_2 assimilation rate (`An_dataVcmax`), the ambient CO_2 partial pressure (`Ca_dataVcmax`), the intercellular CO_2 partial pressure (`Ci_dataVcmax`), and the irradiance (`Iinc_dataVcmax`) . If one wants to estimate V_{cmax} from another dataset, these vectors should be updated. It also contains a variable for calculated rates of linear electron transport (`J_dataVcmax`). The latter one is not used for calculations, because it is assumed in this estimation procedure that the net CO_2 assimilation is limited by Rubisco. However, It should be initialized nevertheless with values. Any combination of values can be chosen, but the length of this vector should equal the length of the other vectors.

If the code is run, a number of *.mat files are generated. These files store input data and output data. The output data for R_d are stored in the *.mat files RdInner.mat, RdGaps.mat, and RdOuter.mat for the scenarios that assumes (photo)respired CO_2 release in the inner cytosol, the cytosol gaps, and the outer cytosol, respectively. The standard deviation of the estimate of R_d for each scenario are stored in the *.mat files stdRdInner.mat, stdRdGaps.mat, and stdRdOuter.mat. Similarly, the estimates of V_{cmax} are stored in VcmaxInner.mat, VcmaxGaps.mat, and VcmaxOuter.mat and the standard deviation of their estimates in stdVcmaxInner.mat, stdVcmaxGaps.mat, and stdVcmaxOuter.mat.

Notes S1.6 Description of M-files

The aim of this section is to describe the function of each M-file and to explain which M-files are run by other M-files. In order to get more explanation on the code within a certain M-file, we recommend to study the comments inside the M-file of interest (Notes S2).

Notes S1.6.1. Main.m

This file is the main function. If this file is run, both V_{cmax} and R_d are estimated for each scenario. In order to do so, it runs estimateRd.m and estimateVcmax.m.

Notes S1.6.2 EstimateRd.m

The M-file estimateRd.m is run by main.m. The function of estimateRd.m file is to obtain estimates for R_d for each scenario. In order to do so, it runs a number of other M-files. Those are initiateModel.m,buildModelInner.m, buildModelOutel.m, buildModelGaps.m, and initiateSolver.m. It also optimizes the function calculateResiduals() that is stored in the M-file calculateResiduals.m.

Notes S1.6.3 EstimateVcmax.m

The M-file estimateVcmax.m is run by main.m. The function of the estimateVcmax.m file is to obtain estimates for V_{cmax} for each scenario. In order to do so, it runs a number of other M-files. Those are initiateModel.m,buildModelInner.m, buildModelOuterl.m, buildModelGaps.m, and initiateSolver.m. It also optimizes the function calculateResiduals() that is stored in the M-file calculateResiduals.m. For each scenario, estimateVcmax.m uses the R_{d} values that were previously estimated by estimateRd.m for that particular scenario as input values for R_{d}.

Notes S1.6.4 InitiateModel.m

The M-file initiateModel.m is run by both estimateRd.m and estimateVcmax.m. The function of initiateModel is to create an empty COMSOL model, define parameters, set the values of these parameters, and generate an empty geometry and mesh. In order to assign the parameters to the empty model, it runs setParameters.m.

Notes S1.6.5 SetParameters.m

The M-file setParameters.m is run by initiateModel.m. The function of setParameters.m is to assign parameters, their values, and their units to an empty COMSOL model. In order to collect the values for the parameters, setParameters.m runs parameters.m.

Notes S1.6.6 Parameters.m

The M-file parameters.m is run by setParameters.m. Parameter.m stores values for each environmental, physiological, and leaf anatomical parameter.

Notes S1.6.7 BuildModelInner.m

The M-file buildModelInner is run by both estimateRd.m and estimateVcmax.m. Its function is to build a COMSOL model for the scenario that assumes (photo)respiratory CO_2 release in the inner cytosol. This means that it uses the parameters that were previously set by setParametes.m to generate a geometry, define additional parameters that were not required to generate the geometry, and to define partial differential equations and their boundary conditions.

Notes S1.6.8 BuildModelOuter.m

The M-file buildModelInner is run by both estimateRd.m and estimateVcmax.m. Its function is to build a COMSOL model for the scenario that assumes (photo)respiratory CO_2 release in the outer cytosol. This means that it uses the parameters that were previously set by setParametes.m to generate a geometry, define additional parameters that were not required to generate the geometry, and to define partial differential equations and their boundary conditions.

Notes S1.6.9 BuildModelGaps.m

The M-file buildModelInner is run by both estimateRd.m and estimateVcmax.m. Its function is to build a COMSOL model for the scenario that assumes (photo)respiratory CO_2 release in the cytosol gaps. This means that it uses the parameters that were previously set by setParametes.m to generate a geometry, to define additional parameters that were not required to generate the geometry, and to define partial differential equations and their boundary conditions.

Notes S1.6.10 CalculateResidualsRd.m

The M-file calculateResiduals.m contains the function calculateResiduals(). The aim of this function is to calculate the residuals (difference between measured and simulated net CO_2 assimilation rates) for a certain set of certain environmental conditions (levels of oxygen, CO_2 and light). This function is optimized in the M-file estimateRd.m in order to find the optimal value of R_d.

Notes S1.6.11 DataRd.m

The M-file dataRd.m is called by CalculateResidualsRd.m. It stores the mean of measured net CO_2 assimilation rates, ambient CO_2 partial pressures, intercellular CO_2 partial pressures and rates of linear electron transports. These measured data are used to estimate R_d.

Notes S1.6.12 CalculateResidualsVcmax.m

The M-file calculateResiduals.m contains the function calculateResiduals(). The aim of this function is to calculate the sum residuals (difference between measured and simulated net CO_2 assimilation rates) for a certain set of certain environmental conditions (levels of oxygen, CO_2 and light). This function is optimized in the M-file estimateRd.m in order to find the optimal value of V_cmax. The optimization is done

by the MATLAB function lsqnonlin(). CalculateResiduals.m obtains measured data to do this optimization by running dataVcmax.m and it obtains simulated data by running the model by running the M-file solveModel.m. It calculates a vector of residuals and calculates the sum of residuals from this vector.

Notes S1.6.13 DataVcmax.m

The M-file dataRd.m is called by CalculateResidualsRd.m. It stores the mean of measured net CO_2 assimilation rates, ambient CO_2 partial pressures, intercellular CO_2 partial pressures and rates of linear electron transports. These measured data are used to estimate R_{d}.

Notes S2: code of M files

Main.m

```
%%%%%%%%%%%%%%%%%%%%%%%%%%%%%%%%%%%%%%%%%%%%%%%%%%%%%%%%%%%%%%%%%%%%%%%%%
%
% Title:    Main.m
%
% Authors:  dr. Herman N.C. Berghuijs
%           dr. Xinyou Yin
%           dr. Quang Tri Ho
%           dr. Moges A. Retta
%           prof. dr. Bart M. Nicolaï
%           prof. dr. Paul C. Struik
%
% Date:     March 11 2019
%
% For any questions regarding the code, please contact Herman Berghuijs
% (herman.berghuijs@slu.se / hermanberghuijs@hotmail.com)
%%%%%%%%%%%%%%%%%%%%%%%%%%%%%%%%%%%%%%%%%%%%%%%%%%%%%%%%%%%%%%%%%%%%%%%%%
%
% The aim of this file is to initiate the procedure to estimate Rd
% from a light response curve measured under low irradiances using a
% reaction diffusion model and to use this estimate as input for another
% procedure to estimate Vcmax. In order to successfully run it, the
% following files have to be present in the work directory:
%
%   - main.m (this file)
%   - buldModelGaps.m
%   - buildModelInner.m
%   - buildModelOuter.m
%   - calculateResidualRd.m
%   - calculateResidualVcmax.m
%   - dataRd.m
%   - dataVcmax.m
%   - estimateRd.m
%   - estimateVcmax.m
%   - initiateModel.m
%   - initiateSolver.m
%   - parameters.m
%   - setParameters.m
%   - solveModel.m
%
% If necessary, the variable currentDirectory (line 49) should be adjusted
% to match the directory in which the user has stored the files listed
% above.
%
%%%%%%%%%%%%%%%%%%%%%%%%%%%%%%%%%%%%%%%%%%%%%%%%%%%%%%%%%%%%%%%%%%%%%%%%%
% Clear memory and console
clear all;
clc;
%
% Set working directory and save it into a mat data file
currentDirectory = 'M:\My Documents\Workdirectory';
cd(currentDirectory)
save currentDirectory currentDirectory;
%
% Start estimation procedure
```

```
display('-------------------------------------------------');
display('Start simulations for 15-day old Admiro leaves');
display('-------------------------------------------------');
%
% Estimate Rd
run([currentDirectory,'\estimateRd.m']);
%
load('currentDirectory.mat')
%
% Use the estimates of Rd as input for the model to estimate Vcmax for each
% scenario.
run([currentDirectory,'\estimateVcmax.m']);
%%%%%%%%%%%%%%%%%%%%%%%%%%%%%%%%%%%%%%%%%%%%%%%%%%%%%%%%%%%%%%%%%%%%%%%%%%
```

EstimateRd.m

```
%%%%%%%%%%%%%%%%%%%%%%%%%%%%%%%%%%%%%%%%%%%%%%%%%%%%%%%%%%%%%%%%%%%%%%%%%
%
% Title:    EstimateRd.m
%
% Authors:  dr. Herman N.C. Berghuijs
%           dr. Xinyou Yin
%           dr. Quang Tri Ho
%           dr. Moges A. Retta
%           prof. dr. Bart M. Nicolaï
%           prof. dr. Paul C. Struik
%
% Date:     March 11 2019
%
% For any questions regarding the code, please contact Herman Berghuijs
% (herman.berghuijs@slu.se / hermanberghuijs@hotmail.com)
%%%%%%%%%%%%%%%%%%%%%%%%%%%%%%%%%%%%%%%%%%%%%%%%%%%%%%%%%%%%%%%%%%%%%%%%%
%
% EstimateRd.m is called by the main function main.m. The aim of this
% M-file is to call each M file in the right sequences that is used to
% estimate Rd from the reaction diffusion model and to store the estimates
% that were obtained into *.MAT datafiles.
%
%%%%%%%%%%%%%%%%%%%%%%%%%%%%%%%%%%%%%%%%%%%%%%%%%%%%%%%%%%%%%%%%%%%%%%%%%
%% 0. Clear memory
%%%%%%%%%%%%%%%%%%%%%%%%%%%%%%%%%%%%%%%%%%%%%%%%%%%%%%%%%%%%%%%%%%%%%%%%%
clear all
%clc
%
%%%%%%%%%%%%%%%%%%%%%%%%%%%%%%%%%%%%%%%%%%%%%%%%%%%%%%%%%%%%%%%%%%%%%%%%%
%% 1. Estimate Rd: CO2 release by (photo)respiration in inner cytosol
%%%%%%%%%%%%%%%%%%%%%%%%%%%%%%%%%%%%%%%%%%%%%%%%%%%%%%%%%%%%%%%%%%%%%%%%%
%
display('Build model for (photo)respired CO2 release in inner cytosol');
%
% Create a COMSOL Multiphysics model, assign parameters to it, and build
% the geometry by running the M-file initiateModel.m.
run('initiateModel.m');
%
% Build the model under the assumption that CO2 produced by
% photorespiration is released in the inner cytosol by running the M-file
% buildModelInner.m.
run('buildModelInner.m');
%
% Initiate the solver by running the M-file initiateSolver.m.
run('initiateSolver.m');
%
% Set the volumetric rate of net CO₂ assimilation equal to the net CO₂
% assimilation rate under electron transport limited conditions.
model.variable('var10').set('w', 'wj');
%
% Generate a COMSOL *.mph file.
model.save([pwd,'\model2']);
%
% Optimize the residuals and calculate the standard error of the estimate
display('Start estimation of Rd');
%
x0=3;                % Set starting value of Rd
```

```
lb=0.1;             % Set lower boundary of the Rd estimate
ub=5;               % Set upper boundary of the Rd estimate
options = optimset('DiffMinChange',1e-4);
%
% Estimate Rd by minimizing the difference between measured net CO2
% assimilation rates and the simulated ones.
[beta,resnorm,resid,exitflag,output,lambda,J]
=lsqnonlin(@calculateResidualRd,x0,lb,ub,options);
%
% Calculate the standard error of the estimate.
sigma=sqrt(resnorm*diag(full((J'*J)^-1)));
%
% Save the estimate of Rd and its standard error in a *.MAT data file
RdInner=beta;
save RdInner RdInner;
stdRdInner=sigma;
save stdRdInner stdRdInner;
%%%%%%%%%%%%%%%%%%%%%%%%%%%%%%%%%%%%%%%%%%%%%%%%%%%%%%%%%%%%%%%%%%%%%%%%%%%
%% 2. Estimate Rd: CO2 release by (photo)respiration in outer cytosol
%%%%%%%%%%%%%%%%%%%%%%%%%%%%%%%%%%%%%%%%%%%%%%%%%%%%%%%%%%%%%%%%%%%%%%%%%%%
%
display('Build model for (photo)respired CO2 release in Outer cytosol');
%
% Create a COMSOL Multiphysics model, assign parameters to it, and build
% the geometry by running the M-file initiateModel.m.
run('initiateModel.m');
%
% Build the model under the assumption that CO2 produced by
% photorespiration is released in the outer cytosol by running the M-file
% buildModelOuter.m.
run('buildModelOuter.m');
%
% Initiate the solver by running initiateSolver.m.
run('initiateSolver.m');
%
% Set the volumetric rate of net CO2 assimilation equal to the net CO2
% assimilation rate under electron transport limited conditions.
model.variable('var10').set('w', 'wj');
model.save([pwd,'\model2']);
display('Start estimation of Rd');
x0=3;
lb=1;
ub=5;
%
% Optimize the residuals and calculate the standard error of the estimate
% by non linear regression.
options = optimset('DiffMinChange',1e-4);
%
% Estimate Rd by minimizing the difference between measured net CO2
% assimilation rates and the simulated ones.
[beta,resnorm,resid,exitflag,output,lambda,J]
=lsqnonlin(@calculateResidualRd,x0,lb,ub,options);
%
% Calculate the standard error of the estimate.
sigma=sqrt(resnorm*diag(full((J'*J)^-1)));
%
% Save the estimate of Rd and its standard error in a *.MAT data file
RdOuter=beta;
save RdOuter RdOuter;
%
stdRdOuter=sigma;
```

```
save stdRdOuter stdRdOuter;
%
%%%%%%%%%%%%%%%%%%%%%%%%%%%%%%%%%%%%%%%%%%%%%%%%%%%%%%%%%%%%%%%%%%%%%%%%%
%% 3. Estimate Rd: CO2 release by (photo)respiration in cytosol gaps
%%%%%%%%%%%%%%%%%%%%%%%%%%%%%%%%%%%%%%%%%%%%%%%%%%%%%%%%%%%%%%%%%%%%%%%%%
display('Build model for (photo)respired CO2 release in cytosol gaps');
%
% Create a COMSOL Multiphysics model, assign parameters to it, and build
% the geometry by running initiateModel.m.
run('initiateModel.m');
%
% Build the model under the assumption that CO2 produced by
% photorespiration is released in the cytosol gaps by running the M-file
% buildModelGaps.m.
run('buildModelGaps.m');
%
% Initiate the solver by running the M-file initiateSolver.m
run('initiateSolver.m');
%
% Set the volumetric rate of net CO2 assimilation equal to the net CO2
% assimilation rate under electron transport limited conditions.
model.variable('var10').set('w', 'wj');
model.save([pwd,'\model2']);
display('Start estimation of Rd');
x0=3;
lb=1;
ub=5;
%
options = optimset('DiffMinChange',1e-4);
%
% Optimize the residuals and calculate the standard error of the estimate
[beta,resnorm,resid,exitflag,output,lambda,J]
=lsqnonlin(@calculateResidualRd,x0,lb,ub,options);
%
% Calculate the standard error of the estimate.
sigma=sqrt(resnorm*diag(full((J'*J)^-1)));
%
% Save the estimate of Rd and its standard error in a *.MAT data file
RdGaps=beta;
save RdGaps RdGaps;
%
stdRdGaps=sigma;
save stdRdGaps stdRdGaps;
%
%%%%%%%%%%%%%%%%%%%%%%%%%%%%%%%%%%%%%%%%%%%%%%%%%%%%%%%%%%%%%%%%%%%%%%%%%%
```

EstimateVcmax.m

```
%%%%%%%%%%%%%%%%%%%%%%%%%%%%%%%%%%%%%%%%%%%%%%%%%%%%%%%%%%%%%%%%%%%%%%%%%%
%
% Title:    EstimateVcmax.m
%
% Authors:  dr. Herman N.C. Berghuijs
%           dr. Xinyou Yin
%           dr. Quang Tri Ho
%           dr. Moges A. Retta
%           prof. dr. Bart M. Nicolaï
%           prof. dr. Paul C. Struik
%
% Date:     March 11 2019
%
% For any questions regarding the code, please contact Herman Berghuijs
% (herman.berghuijs@slu.se / hermanberghuijs@hotmail.com)
%%%%%%%%%%%%%%%%%%%%%%%%%%%%%%%%%%%%%%%%%%%%%%%%%%%%%%%%%%%%%%%%%%%%%%%%%%
%
% EstimateVcmax.m is called by the main function main.m. The aim of this
% M-file is to call each M file in the right sequences that is used to
% estimate Vcmax from the reaction diffusion model and to store the
% estimates that were obtained into *.MAT datafiles. It is called by the
% M-file main.m after estimateRd.m is called such that the estimates of Rd
% for each scenario are loaded into this script.
%
%%%%%%%%%%%%%%%%%%%%%%%%%%%%%%%%%%%%%%%%%%%%%%%%%%%%%%%%%%%%%%%%%%%%%%%%%%
%% 0. Clear memory
%%%%%%%%%%%%%%%%%%%%%%%%%%%%%%%%%%%%%%%%%%%%%%%%%%%%%%%%%%%%%%%%%%%%%%%%%%
%
clear all
%
%%%%%%%%%%%%%%%%%%%%%%%%%%%%%%%%%%%%%%%%%%%%%%%%%%%%%%%%%%%%%%%%%%%%%%%%%%
%% 1. Estimate Vcmax: CO2 release by (photo)respiration in inner cytosol
%%%%%%%%%%%%%%%%%%%%%%%%%%%%%%%%%%%%%%%%%%%%%%%%%%%%%%%%%%%%%%%%%%%%%%%%%%
%
% Load the Rd estimate that was previously obtained for the scenario of
% (photo)respired CO2 release in the inner cytosol.
load('RdInner.mat');
display('Build model for (photo)respired CO2 release in inner cytosol');
%
% Create a COMSOL Multiphysics model, assign parameters to it, and build
% the geometry by running the M-file initiateModel.m.
initiateModel;
%
% Build the model under the assumption that CO2 produced by
% photorespiration is released in the inner cytosol by running the M-file
% buildModelInner.m.
buildModelInner;
%
% Initiate the solver by running the M-file initiateSolver.m.
initiateSolver;
%
% Set the volumetric rate of net CO2 assimilation equal to the net CO2
% assimilation rate under Rubisco transport limited conditions.
model.variable('var10').set('w', 'wc');
%
% Set Rd equal to the estimate that was previously obtained for the
% scenario that assumes (photo)respired CO2 release in the inner cytosol.
```

```
model.param.set('Rd', [num2str(RdInner),'[umol][m^-2][s^-1]']);
%
% Generate a COMSOL *.mph file.
model.save([pwd,'\model2']);
display('Start estimation of Vcmax');
x0=100;              % Set starting value of Vcmax
lb=25;               % Set lower boundary of the Vcmax estimate
ub=400;              % Set upper boundary of the Vcmax estimate
options = optimset('DiffMinChange',1e-4);
%
% Estimate Vcmax by minimizing the difference between measured net CO2
% assimilation rates and the simulated ones.
[beta,resnorm,resid,exitflag,output,lambda,jacobian]
=lsqnonlin(@calculateResidualVcmax,x0,lb,ub,options);
%
% Calculate the standard error of the estimate.
sigma=sqrt(resnorm*diag(full((jacobian'*jacobian)^-1)));
%
% Save the estimate of Vcmax and its standard error in a *.MAT data file
VcmaxInner=beta;
save VcmaxInner VcmaxInner;
stdVcmaxInner=sigma;
save stdVcmaxInner stdVcmaxInner;
%
%%%%%%%%%%%%%%%%%%%%%%%%%%%%%%%%%%%%%%%%%%%%%%%%%%%%%%%%%%%%%%%%%%%%%%%%%%
%% 2. Estimate Vcmax: CO2 release by (photo)respiration in outer cytosol
%%%%%%%%%%%%%%%%%%%%%%%%%%%%%%%%%%%%%%%%%%%%%%%%%%%%%%%%%%%%%%%%%%%%%%%%%%
% Load the Rd estimate that was previously obtained for the scenario of
% (photo)respired CO2 release in the outer cytosol.
load('RdOuter.mat')
%
display('Build model for (photo)respired CO2 release in Outer cytosol');
%
% Create a COMSOL Multiphysics model, assign parameters to it, and build
% the geometry by running the M-file initiateModel.m.
initiateModel;
%
% Build the model under the assumption that CO2 produced by
% photorespiration is released in the outer cytosol by running the M-file
% buildModelInner.m.
buildModelOuter;
%
% Initiate the solver by running the M-file initiateSolver.m.
initiateSolver;
%
% Set the volumetric rate of net CO2 assimilation equal to the net CO2
% assimilation rate under Rubisco transport limited conditions.
model.variable('var10').set('w', 'wc');
% Set Rd equal to the estimate that was previously obtained for the
% scenario that assumes (photo)respired CO2 release in the outer cytosol.
model.param.set('Rd', [num2str(RdOuter),'[umol][m^-2][s^-1]']);
model.save([pwd,'\model2']);
%
display('Start estimation of Vcmax');
%
% Estimate Vcmax by minimizing the difference between measured net CO2
% assimilation rates and the simulated ones.
x0=100;
lb=25;
ub=400;
options = optimset('DiffMinChange',1e-4);
```

```
%
% Estimate Vcmax by minimizing the difference between measured net CO2
% assimilation rates and the simulated ones.
[beta,resnorm,resid,exitflag,output,lambda,jacobian]
=lsqnonlin(@calculateResidualVcmax,x0,lb,ub,options);
%
% Calculate the standard error of the estimate.
sigma=sqrt(resnorm*diag(full((jacobian'*jacobian)^-1)));
%
% Save the estimate of Vcmax and its standard error in a *.MAT data file
VcmaxOuter=beta;
save VcmaxOuter VcmaxOuter;
stdVcmaxOuter=sigma;
save stdVcmaxOuter stdVcmaxOuter;
%%%%%%%%%%%%%%%%%%%%%%%%%%%%%%%%%%%%%%%%%%%%%%%%%%%%%%%%%%%%%%%%%%%%%%%%%
%% 3. Estimate Vcmax: CO2 release by (photo)respiration in cytosol gaps
%%%%%%%%%%%%%%%%%%%%%%%%%%%%%%%%%%%%%%%%%%%%%%%%%%%%%%%%%%%%%%%%%%%%%%%%%
% Load the Rd estimate that was previously obtained for the scenario of
% (photo)respired CO2 release in the outer cytosol.
load RdGaps
%
display('Build model for (photo)respired CO2 release in cytosol gaps');
%
% Build the model under the assumption that CO2 produced by
% photorespiration is released in the cytosol gaps by running the M-file
% buildModelInner.m.
initiateModel;
%
% Initiate the solver by running the M-file initiateSolver.m.
buildModelGaps;
%
% Initiate the solver by running the M-file initiateSolver.m.
initiateSolver;
%
% Set the volumetric rate of net CO2 assimilation equal to the net CO2
% assimilation rate under Rubisco transport limited conditions.
model.variable('var10').set('w', 'wc');
% Set Rd equal to the estimate that was previously obtained for the
% scenario that assumes (photo)respired CO2 release in the cytosol gaps.
model.param.set('Rd', [num2str(RdGaps),'[umol][m^-2][s^-1]']);
model.save([pwd,'\model2']);
%
% Estimate Vcmax by minimizing the difference between measured net CO2
% assimilation rates and the simulated ones.
display('Start estimation of Vcmax');
x0=100;
lb=25;
ub=400;
options = optimset('DiffMinChange',1e-4);
[beta,resnorm,resid,exitflag,output,lambda,jacobian]
=lsqnonlin(@calculateResidualVcmax,x0,lb,ub,options);
%
% Calculate the standard error of the estimate.
sigma=sqrt(resnorm*diag(full((jacobian'*jacobian)^-1)));
%
% Save the estimate of Vcmax and its standard error in a *.MAT data file
VcmaxGaps=beta;
save VcmaxGaps VcmaxGaps;
stdVcmaxGaps=sigma;
save stdVcmaxGaps stdVcmaxGaps;
%%%%%%%%%%%%%%%%%%%%%%%%%%%%%%%%%%%%%%%%%%%%%%%%%%%%%%%%%%%%%%%%%%%%%%%%%
```

InitiateModel.m

```
%%%%%%%%%%%%%%%%%%%%%%%%%%%%%%%%%%%%%%%%%%%%%%%%%%%%%%%%%%%%%%%%%%%%%%%%%%
%
% Title:    initiateModel.m
%
% Authors:  dr. Herman N.C. Berghuijs
%           dr. Xinyou Yin
%           dr. Quang Tri Ho
%           dr. Moges A. Retta
%           prof. dr. Bart M. Nicolaï
%           prof. dr. Paul C. Struik
%
% Date:     March 11 2019
%
% For any questions regarding the code, please contact Herman Berghuijs
% (herman.berghuijs@slu.se / hermanberghuijs@hotmail.com)
%%%%%%%%%%%%%%%%%%%%%%%%%%%%%%%%%%%%%%%%%%%%%%%%%%%%%%%%%%%%%%%%%%%%%%%%%%
%
% The aim of this m file is to initialize the model. It creates an empty
% COMSOL model, assigns parameters to it and defines an empty mesh and an
% empty geometry. This file is called by the M-file estimateRd2.m.
%
%%%%%%%%%%%%%%%%%%%%%%%%%%%%%%%%%%%%%%%%%%%%%%%%%%%%%%%%%%%%%%%%%%%%%%%%%%
%
% Load COMSOL memories to link COMSOL with MATLAB
import com.comsol.model.*
import com.comsol.model.util.*
%
% Create a model
model = ModelUtil.create('Model');
%
% Assign physiological, anatomical and environmental parameters to the
% model.
run('setParameters.m')
%
% Further built the model and define an empty geometry and an empty mesh.
model.modelNode.create('comp1');
model.file.clear;
model.geom.create('geom1', 2);
model.mesh.create('mesh1', 'geom1');
%%%%%%%%%%%%%%%%%%%%%%%%%%%%%%%%%%%%%%%%%%%%%%%%%%%%%%%%%%%%%%%%%%%%%%%%%%%
```

SetParameters.m

```
%%%%%%%%%%%%%%%%%%%%%%%%%%%%%%%%%%%%%%%%%%%%%%%%%%%%%%%%%%%%%%%%%%%%%%%%%%
%
% Title:    setParameters.m
%
% Authors:  dr. Herman N.C. Berghuijs
%           dr. Xinyou Yin
%           dr. Quang Tri Ho
%           dr. Moges A. Retta
%           prof. dr. Bart M. Nicolaï
%           prof. dr. Paul C. Struik
%
% Date:     March 11 2019
%
% For any questions regarding the code, please contact Herman Berghuijs
% (herman.berghuijs@slu.se / hermanberghuijs@hotmail.com)
%%%%%%%%%%%%%%%%%%%%%%%%%%%%%%%%%%%%%%%%%%%%%%%%%%%%%%%%%%%%%%%%%%%%%%%%%%
%
% The aim of this M-file is to assign parameters to the model. The values
% of the parameters are loaded by running parameters.m and subsequently
% assigned to the model. Finally, it calculates the parameters h_GAP and
% h_STR. These parameters have to be calculated before the model is solved,
% because these parameters are essential to build the geometry.
%
%%%%%%%%%%%%%%%%%%%%%%%%%%%%%%%%%%%%%%%%%%%%%%%%%%%%%%%%%%%%%%%%%%%%%%%%%%
%
% Run the M-file parameters.m to define the model parameters.
run('parameters.m');
%
% Assign the model parameters to the COMSOL model.
model.param.set('t_CYT', [num2str(t_CYT),'[m]']);
model.param.set('t_STR', [num2str(t_STR),'[m]']);
model.param.set('Sm_S', num2str(Sm_S));
model.param.set('t_WALL', [num2str(t_WALL),'[m]']);
model.param.set('Sc_Sm', num2str(Sc_Sm));
model.param.set('ratio', num2str(ratio));
model.param.set('Iinc0', [num2str(Iinc),'[umol][m^-2][s^-1]']);
model.param.set('Ci', [num2str(Ci),'[Pa]']);
model.param.set('O', [num2str(O),'[kPa]']);
model.param.set('Vcmax', [num2str(Vcmax),'[umol][m^-2][s^-1]']);
model.param.set('Rd', [num2str(Rd),'[umol][m^-2][s^-1]']);
model.param.set('Tp', [num2str(Tp),'[umol][m^-2][s^-1]']);
model.param.set('ScO', [num2str(ScO),'[mbar][ubar^-1]']);
model.param.set('KmC',[num2str(KmC),'[Pa]']);
model.param.set('KmO',[num2str(KmO),'[kPa]']);
%
% Calculate the parameters h_STR and h_GAP and assign them to the COMSOL
% model.
model.param.set('h_STR', 'ratio*t_STR');
model.param.set('h_GAP', 'ratio*t_STR*((Sc_Sm^-1)-1)');
%%%%%%%%%%%%%%%%%%%%%%%%%%%%%%%%%%%%%%%%%%%%%%%%%%%%%%%%%%%%%%%%%%%%%%%%%%
```

Parameters.m

```
%%%%%%%%%%%%%%%%%%%%%%%%%%%%%%%%%%%%%%%%%%%%%%%%%%%%%%%%%%%%%%%%%%%%%%%%%
%
% Title:    Parameters.m
%
% Authors:  dr. Herman N.C. Berghuijs
%           dr. Xinyou Yin
%           dr. Quang Tri Ho
%           dr. Moges A. Retta
%           prof. dr. Bart M. Nicolaï
%           prof. dr. Paul C. Struik
%
% Date:     March 11 2019
%
% For any questions regarding the code, please contact
%%%%%%%%%%%%%%%%%%%%%%%%%%%%%%%%%%%%%%%%%%%%%%%%%%%%%%%%%%%%%%%%%%%%%%%%%
%
% This file initiates leaf anatomical, physiological and environmental
% parameters, except Rd (which is the parameter to be estimated). It is
% called by the M-file setParameters.m.
%
%%%%%%%%%%%%%%%%%%%%%%%%%%%%%%%%%%%%%%%%%%%%%%%%%%%%%%%%%%%%%%%%%%%%%%%%%

%% Set anatomical parameters
t_CYT=243e-9;       % Cytosol thickness (m);
t_STR=2.5458e-6;    % Stroma thickness (m);
Sm_S=17;            % Ratio of the mesophyll surface area exposed to
                    % the intercellular airspaces to leaf surface area
t_WALL=118e-9;      % Cell wall thickness (m)
Sc_Sm=0.919;        % Ratio of the area of the chloroplast surface facing
                    % the intercellular airspace to the mesophyll surface
                    % area exposed to the intercellular air space.
ratio=2.5;          % Ratio stroma thickness : stroma height

%% Set environmental parameters
Iinc=1500;         % Irradiance (umol m^-2 s^-1)
Ci=25;              % CO2 partial pressure in intercellular air spaces (Pa)
O=21;               % Oxygen partial pressure (kPa)

%% Set physiological parameters
Vcmax=256;          % Maximum rate of RuBP carboxylation (umol m^-2 s^-1)
ScO=2.6;            % Rubisco specificity (mbar ubar^-1)
Rd=2.46;            % Rate of respiration (umol m^-2 s^-1)
Tp=12.8;            % Rate of triose phosphate utilization (umol m^-2 s^-1)
KmC=26.4;           % Michaelis Menten constant for RuBP carboxylation (Pa)
KmO=16.4;           % Michaelis Menten constant for RuBP oxygenation (kPa);
J=100;              % Linear rate of electron transport (umol m-2 s-1)
%
%%%%%%%%%%%%%%%%%%%%%%%%%%%%%%%%%%%%%%%%%%%%%%%%%%%%%%%%%%%%%%%%%%%%%%%%%%%
```

buildModelInner.m

```
%%%%%%%%%%%%%%%%%%%%%%%%%%%%%%%%%%%%%%%%%%%%%%%%%%%%%%%%%%%%%%%%%%%%%%%%%
%
% Title:    buildModelOuter.m
%
% Authors:  dr. Herman N.C. Berghuijs
%           dr. Xinyou Yin
%           dr. Quang Tri Ho
%           dr. Moges A. Retta
%           prof. dr. Bart M. Nicolaï
%           prof. dr. Paul C. Struik
%
% Date:     March 11 2019
%
% For any questions regarding the code, please contact Herman Berghuijs
% (herman.berghuijs@slu.se / hermanberghuijs@hotmail.com)
%%%%%%%%%%%%%%%%%%%%%%%%%%%%%%%%%%%%%%%%%%%%%%%%%%%%%%%%%%%%%%%%%%%%%%%%%
%
% This file implements model equations, generates the geometry, defines
% the different subdomains within the geometry, and builds the mesh. In
% this M-file, it is assumed that CO2 produced by respiration and
% photorespiration is released in the inner cytosol.
%
%%%%%%%%%%%%%%%%%%%%%%%%%%%%%%%%%%%%%%%%%%%%%%%%%%%%%%%%%%%%%%%%%%%%%%%%%
%
% Define and built the model geometry
model.geom('geom1').create('r1', 'Rectangle');
model.geom('geom1').feature('r1').setIndex('layer', 't_CYT', 0);
model.geom('geom1').feature('r1').set('layerbottom', false);
model.geom('geom1').feature('r1').set('size', {'t_STR+2*t_CYT'
'0.5*(h_GAP+h_STR)'});
model.geom('geom1').feature('r1').set('layername', {'Layer 1'});
model.geom('geom1').create('r2', 'Rectangle');
model.geom('geom1').feature('r2').set('layerbottom', false);
model.geom('geom1').feature('r2').set('size', {'t_STR'
'0.5*(h_STR+h_GAP)'});
model.geom('geom1').feature('r2').set('pos', {'t_CYT' '0'});
model.geom('geom1').create('r3', 'Rectangle');
model.geom('geom1').feature('r3').set('layer', {'t_CYT' 't_STR' 't_CYT'});
model.geom('geom1').feature('r3').set('layerbottom', false);
model.geom('geom1').feature('r3').set('size', {'t_STR+2*t_CYT'
'0.5*h_GAP'});
model.geom('geom1').feature('r3').set('pos', {'0' '0'});
model.geom('geom1').feature('r3').set('layername', {'Layer 1' 'Layer 1' ''
''});
model.geom('geom1').run;
%
% Define the different subdomains of the geometry.
model.selection.create('sel1', 'Explicit');
model.selection('sel1').set([5 6]);
model.selection.create('sel2', 'Explicit');
model.selection('sel2').set([1 2]);
model.selection.create('sel3', 'Explicit');
model.selection('sel3').set([3]);
model.selection.create('sel4', 'Explicit');
model.selection('sel4').set([4]);
model.selection.create('sel5', 'Explicit');
model.selection('sel5').geom('geom1', 1);
```

```
model.selection('sel5').set([1 3]);
model.selection.create('sel6', 'Explicit');
model.selection('sel6').geom('geom1', 1);
model.selection('sel6').set([16 17]);
model.selection.create('uni1', 'Union');
model.selection.create('uni2', 'Union');
model.selection.create('uni3', 'Union');
model.selection.create('uni4', 'Union');
model.selection.create('sel8', 'Explicit');
model.selection('sel8').geom('geom1', 1);
model.selection.create('uni5', 'Union');
model.selection.create('sel10', 'Explicit');
model.selection('sel10').geom('geom1', 1);
model.selection('sel10').set([8 9 13]);
model.selection('sel1').label('Inner cytosol');
model.selection('sel1').label('Explicit 1');
model.selection('sel2').label('Outer cytosol');
model.selection('sel2').label('Explicit 2');
model.selection('sel3').label('Cytosol gap');
model.selection('sel3').label('Explicit 3');
model.selection('sel4').label('Stroma');
model.selection('sel4').label('Explicit 4');
model.selection('sel5').label('Cell wall and plasma membrane');
model.selection('sel5').label('Explicit 5');
model.selection('sel6').label('Tonoplast');
model.selection('sel6').label('Explicit 6');
model.selection('uni1').set('input', {'sel1' 'sel2' 'sel3'});
model.selection('uni1').label('Cytosol');
model.selection('uni2').set('input', {'sel1' 'sel4'});
model.selection('uni2').label('Stroma and inner cytosol');
model.selection('uni3').set('input', {'sel2' 'sel4'});
model.selection('uni3').label('Stroma and outer cytosol');
model.selection('uni4').set('input', {'sel4' 'uni1'});
model.selection('uni4').label('Liquid phase');
model.selection('sel8').label('Stomata');
model.selection('sel8').label('Explicit 8');
model.selection('uni5').set('input', {'sel1' 'sel2'});
model.selection('uni5').label('Inner and outer cytosol');
model.selection('sel10').label('Chloroplast envelope');
model.selection('sel10').label('Explicit 10');
%
% Implement model equations and diffusive properties
model.variable.create('var15');
model.variable('var15').model('comp1');
model.variable('var15').set('h_STR', 'ratio*t_STR');
model.variable('var15').set('h_GAP', 'ratio*t_STR*((Sc_Sm^-1)-1)');
model.variable.create('var1');
model.variable('var1').model('comp1');
model.variable('var1').set('S_Vc', '(1/t_STR)*(Sm_S^-1)*(Sc_Sm^-1)');
model.variable('var1').set('S_Vcyt1', '(1/t_CYT)*(Sm_S^-1)');
model.variable('var1').set('S_Vcyt12', '(1/(2*t_CYT))*(Sm_S^-1)');
model.variable('var1').set('S_Vcyt', 'S_Vcyt1');
model.variable('var1').set('Vc_Vc1',
'((2*t_CYT+t_STR)*Sm_S*Sc_Sm)/(ratio*t_STR)');
model.variable('var1').set('l_S', '1/(2*t_CYT+t_STR)');
model.variable.create('var2');
model.variable('var2').model('comp1');
model.variable('var2').set('Gamma', '0.5*O/ScO');
model.variable.create('var3');
model.variable('var3').model('comp1');
model.variable('var3').set('p_eff_WALL', '0.2');
```

```
model.variable('var3').set('zeta_STR', '0.5');
model.variable('var3').set('zeta_CYT', '0.5');
model.variable('var3').set('G_MEM', '3.5e-3[m][s^-1]');
model.variable('var3').set('DCO2_WATER', '1.79e-9[m^2][s^-1]');
model.variable('var3').set('DHCO3_WATER', '0.52*DCO2_WATER');
model.variable('var3').set('DCO2_WALL', 'p_eff_WALL*DCO2_WATER');
model.variable('var3').set('DCO2_CYT', 'zeta_CYT*DCO2_WATER');
model.variable('var3').set('DCO2_STR', 'zeta_STR*DCO2_WATER');
model.variable.create('var4');
model.variable('var4').model('comp1');
model.variable('var4').set('R', '8.314[Pa][m^3][mol^-1][K^-1]');
model.variable('var4').set('H', '2941[Pa][m^3][mol^-1]');
model.variable('var4').set('kH', 'R*T/H');
model.variable('var4').set('T', '298.15[K]');
model.variable.create('var5');
model.variable('var5').model('comp1');
model.variable('var5').set('j_', 'J*S_Vc');
model.variable('var5').set('Iinc', 'Iinc0');
model.variable.create('var6');
model.variable('var6').model('comp1');
model.variable('var6').set('rd', 'Rd*S_Vcyt');
model.variable('var6').set('tp', 'Tp*S_Vc');
model.variable('var6').set('vcmax', 'Vcmax*S_Vc');
model.variable('var6').set('o', 'O/(R*T)');
model.variable('var6').set('gamma', 'kH*Gamma/(R*T)');
model.variable('var6').set('kmc', 'kH*KmC/(R*T)');
model.variable('var6').set('kmo', 'KmO/(R*T)');
model.variable('var6').set('ci', 'Ci/(R*T)');
model.variable.create('var10');
model.variable('var10').model('comp1');
model.variable('var10').set('wc', 'c*vcmax/(c+kmc*(1+o/kmo))');
model.variable('var10').set('wj', 'c*j_/(4*c+8*gamma)');
model.variable('var10').set('wp', '3*tp/(max(eps,1-(gamma/c)))');
model.variable('var10').set('w', 'min(wc,min(wj,wp))');
model.variable.create('var9');
model.variable('var9').model('comp1');
model.variable('var9').set('W_1', 'intop1(w)*l_S');
model.variable('var9').set('F_1', 'intop1(w*gamma/c)*l_S');
model.variable('var9').set('F', '2*F_1*Vc_Vc1');
model.variable('var9').set('W', '2*W_1*Vc_Vc1');
model.variable.create('var11');
model.variable('var11').model('comp1');
model.variable('var11').set('R_MEM', '1/G_MEM');
model.variable('var11').set('G_WALL', 'DCO2_WALL/t_WALL');
model.variable('var11').set('R_WALL', '1/G_WALL');
model.variable('var11').set('R_WP', 'R_WALL+R_MEM');
model.variable('var11').set('G_WP', '1/R_WP');
model.variable.create('var16');
model.variable('var16').model('comp1');
model.variable('var16').set('cc', 'intop1(c)/intop1(1)');
model.variable('var16').set('cc_gas', 'cc/kH');
model.variable('var16').set('Cc', 'cc_gas*R*T');
model.variable('var16').set('An', 'W-F-Rd');
model.variable('var16').set('gm', 'An/(Ci-Cc)');
%
model.view.create('view2', 'geom1');
%
% Define a number of domains over which the model will calculate integrals.
model.cpl.create('intop1', 'Integration', 'geom1');
model.cpl.create('intop2', 'Integration', 'geom1');
model.cpl.create('intop3', 'Integration', 'geom1');
```

```
model.cpl.create('intop4', 'Integration', 'geom1');
model.cpl('intop1').selection.set([4]);
model.cpl('intop2').selection.set([1 2 3 5 6]);
model.cpl('intop3').selection.named('sel4');
model.cpl('intop4').selection.named('sel1');
%
% Define the partial differential equations and the boundary conditions
model.physics.create('tds', 'DilutedSpecies', 'geom1');
model.physics('tds').selection.named('uni4');
model.physics('tds').create('reac1', 'Reactions', 2);
model.physics('tds').feature('reac1').selection.named('sel1');
model.physics('tds').create('reac2', 'Reactions', 2);
model.physics('tds').feature('reac2').selection.named('sel4');
model.physics('tds').create('cdm2', 'ConvectionDiffusionMigration', 2);
model.physics('tds').feature('cdm2').selection.named('sel4');
model.physics('tds').create('cdm3', 'ConvectionDiffusionMigration', 2);
model.physics('tds').feature('cdm3').selection.named('uni1');
model.physics('tds').create('reac3', 'Reactions', 2);
model.physics('tds').feature('reac3').selection.named('sel1');
model.physics('tds').create('fl1', 'Fluxes', 1);
model.physics('tds').feature('fl1').selection.named('sel5');
model.physics('tds').create('tdb1', 'ThinDiffusionBarrier', 1);
model.physics('tds').feature('tdb1').selection.named('sel10');
%
% Assign names to the integrals.
model.cpl('intop1').label('Integrate of stroma');
model.cpl('intop2').label('Integrate over cytosol');
%
% Define the partial differential equations and the boundary conditions
model.physics('tds').label('Transport of C12');
model.physics('tds').prop('TransportMechanism').set('Convection', '0');
model.physics('tds').feature('cdm1').set('D_c', {'DCO2_CYT'; '0'; '0'; '0';
'DCO2_CYT'; '0'; '0'; '0'; 'DCO2_CYT'});
model.physics('tds').feature('init1').set('initc', 'ci*R*T/H');
model.physics('tds').feature('reac1').set('R_c', 'rd');
model.physics('tds').feature('reac1').label('Day respiration');
model.physics('tds').feature('reac2').set('R_c', '-w');
model.physics('tds').feature('reac2').label('CO2 assimilation');
model.physics('tds').feature('cdm2').set('D_c', {'DCO2_STR'; '0'; '0'; '0';
'DCO2_STR'; '0'; '0'; '0'; 'DCO2_STR'});
model.physics('tds').feature('cdm2').label('Diffusion in stroma');
model.physics('tds').feature('cdm3').set('D_c', {'DCO2_CYT'; '0'; '0'; '0';
'DCO2_CYT'; '0'; '0'; '0'; 'DCO2_CYT'});
model.physics('tds').feature('cdm3').label('Diffusion in cytosol');
model.physics('tds').feature('reac3').set('R_c',
'intop3(gamma*w/c)/intop4(1)');
model.physics('tds').feature('reac3').label('Photorespiration');
model.physics('tds').feature('fl1').set('FluxType',
'ExternalForcedConvection');
model.physics('tds').feature('fl1').set('species', '1');
model.physics('tds').feature('fl1').set('kc', 'G_WP');
model.physics('tds').feature('fl1').set('cb', 'ci*kH');
model.physics('tds').feature('fl1').label('Diffusion over cell wall and
plasma membrane');
model.physics('tds').feature('tdb1').set('ds', '1');
model.physics('tds').feature('tdb1').set('Ds', '0.5*3.5e-3');
model.physics('tds').feature('tdb1').label('Diffusion over chloroplast
enveloppe');
%
% Generate the mesh
model.mesh('mesh1').run;
```

%%

buildModelOuter.m

```
%%%%%%%%%%%%%%%%%%%%%%%%%%%%%%%%%%%%%%%%%%%%%%%%%%%%%%%%%%%%%%%%%%%%%%%%%
%
% Title:    buildModelOuter.m
%
% Authors:  dr. Herman N.C. Berghuijs
%           dr. Xinyou Yin
%           dr. Quang Tri Ho
%           dr. Moges A. Retta
%           prof. dr. Bart M. Nicolaï
%           prof. dr. Paul C. Struik
%
% Date:     March 11 2019
%
% For any questions regarding the code, please contact Herman Berghuijs
% (herman.berghuijs@slu.se / hermanberghuijs@hotmail.com)
%%%%%%%%%%%%%%%%%%%%%%%%%%%%%%%%%%%%%%%%%%%%%%%%%%%%%%%%%%%%%%%%%%%%%%%%%
%
% This file implements model equations, generates the geometry, defines
% the different subdomains within the geometry, and builds the mesh. In
% this M-file, it is assumed that CO2 produced by respiration and
% photorespiration is released in the outer cytosol.
%
%%%%%%%%%%%%%%%%%%%%%%%%%%%%%%%%%%%%%%%%%%%%%%%%%%%%%%%%%%%%%%%%%%%%%%%%%
%
% Define and built the model geometry
model.geom('geom1').create('r1', 'Rectangle');
model.geom('geom1').feature('r1').setIndex('layer', 't_CYT', 0);
model.geom('geom1').feature('r1').set('layerbottom', false);
model.geom('geom1').feature('r1').set('size', {'t_STR+2*t_CYT'
'0.5*(h_GAP+h_STR)'});
model.geom('geom1').feature('r1').set('layername', {'Layer 1'});
model.geom('geom1').create('r2', 'Rectangle');
model.geom('geom1').feature('r2').set('layerbottom', false);
model.geom('geom1').feature('r2').set('size', {'t_STR'
'0.5*(h_STR+h_GAP)'});
model.geom('geom1').feature('r2').set('pos', {'t_CYT' '0'});
model.geom('geom1').create('r3', 'Rectangle');
model.geom('geom1').feature('r3').set('layer', {'t_CYT' 't_STR' 't_CYT'});
model.geom('geom1').feature('r3').set('layerbottom', false);
model.geom('geom1').feature('r3').set('size', {'t_STR+2*t_CYT'
'0.5*h_GAP'});
model.geom('geom1').feature('r3').set('pos', {'0' '0'});
model.geom('geom1').feature('r3').set('layername', {'Layer 1' 'Layer 1' ''
''});
model.geom('geom1').run;
%
% Define the different subdomains of the geometry.
model.selection.create('sel1', 'Explicit');
model.selection('sel1').set([5 6]);
model.selection.create('sel2', 'Explicit');
model.selection('sel2').set([1 2]);
model.selection.create('sel3', 'Explicit');
model.selection('sel3').set([3]);
model.selection.create('sel4', 'Explicit');
model.selection('sel4').set([4]);
model.selection.create('sel5', 'Explicit');
model.selection('sel5').geom('geom1', 1);
```

```
model.selection('sel5').set([1 3]);
model.selection.create('sel6', 'Explicit');
model.selection('sel6').geom('geom1', 1);
model.selection('sel6').set([16 17]);
model.selection.create('uni1', 'Union');
model.selection.create('uni2', 'Union');
model.selection.create('uni3', 'Union');
model.selection.create('uni4', 'Union');
model.selection.create('sel8', 'Explicit');
model.selection('sel8').geom('geom1', 1);
model.selection.create('uni5', 'Union');
model.selection.create('sel10', 'Explicit');
model.selection('sel10').geom('geom1', 1);
model.selection('sel10').set([8 9 13]);
model.selection('sel1').label('Inner cytosol');
model.selection('sel1').label('Explicit 1');
model.selection('sel2').label('Outer cytosol');
model.selection('sel2').label('Explicit 2');
model.selection('sel3').label('Cytosol gap');
model.selection('sel3').label('Explicit 3');
model.selection('sel4').label('Stroma');
model.selection('sel4').label('Explicit 4');
model.selection('sel5').label('Cell wall and plasma membrane');
model.selection('sel5').label('Explicit 5');
model.selection('sel6').label('Tonoplast');
model.selection('sel6').label('Explicit 6');
model.selection('uni1').set('input', {'sel1' 'sel2' 'sel3'});
model.selection('uni1').label('Cytosol');
model.selection('uni2').set('input', {'sel1' 'sel4'});
model.selection('uni2').label('Stroma and inner cytosol');
model.selection('uni3').set('input', {'sel2' 'sel4'});
model.selection('uni3').label('Stroma and outer cytosol');
model.selection('uni4').set('input', {'sel4' 'uni1'});
model.selection('uni4').label('Liquid phase');
model.selection('sel8').label('Stomata');
model.selection('sel8').label('Explicit 8');
model.selection('uni5').set('input', {'sel1' 'sel2'});
model.selection('uni5').label('Inner and outer cytosol');
model.selection('sel10').label('Chloroplast envelope');
model.selection('sel10').label('Explicit 10');
%
% Define the different subdomains of the geometry.
model.variable.create('var15');
model.variable('var15').model('comp1');
model.variable('var15').set('h_STR', 'ratio*t_STR');
model.variable('var15').set('h_GAP', 'ratio*t_STR*((Sc_Sm^-1)-1)');
model.variable.create('var1');
model.variable('var1').model('comp1');
model.variable('var1').set('S_Vc', '(1/t_STR)*(Sm_S^-1)*(Sc_Sm^-1)');
model.variable('var1').set('S_Vcyt1', '(1/t_CYT)*(Sm_S^-1)');
model.variable('var1').set('S_Vcyt12', '(1/(2*t_CYT))*(Sm_S^-1)');
model.variable('var1').set('S_Vcyt', 'S_Vcyt1');
model.variable('var1').set('Vc_Vc1',
'((2*t_CYT+t_STR)*Sm_S*Sc_Sm)/(ratio*t_STR)');
model.variable('var1').set('l_S', '1/(2*t_CYT+t_STR)');
model.variable.create('var2');
model.variable('var2').model('comp1');
model.variable('var2').set('Gamma', '0.5*O/ScO');
model.variable.create('var3');
model.variable('var3').model('comp1');
model.variable('var3').set('p_eff_WALL', '0.2');
```

```
model.variable('var3').set('zeta_STR', '0.5');
model.variable('var3').set('zeta_CYT', '0.5');
model.variable('var3').set('G_MEM', '3.5e-3[m][s^-1]');
model.variable('var3').set('DCO2_WATER', '1.79e-9[m^2][s^-1]');
model.variable('var3').set('DHCO3_WATER', '0.52*DCO2_WATER');
model.variable('var3').set('DCO2_WALL', 'p_eff_WALL*DCO2_WATER');
model.variable('var3').set('DCO2_CYT', 'zeta_CYT*DCO2_WATER');
model.variable('var3').set('DCO2_STR', 'zeta_STR*DCO2_WATER');
model.variable.create('var4');
model.variable('var4').model('comp1');
model.variable('var4').set('R', '8.314[Pa][m^3][mol^-1][K^-1]');
model.variable('var4').set('H', '2941[Pa][m^3][mol^-1]');
model.variable('var4').set('kH', 'R*T/H');
model.variable('var4').set('T', '298.15[K]');
model.variable.create('var5');
model.variable('var5').model('comp1');
model.variable('var5').set('j_', 'J*S_Vc');
model.variable('var5').set('Iinc', 'Iinc0');
model.variable.create('var6');
model.variable('var6').model('comp1');
model.variable('var6').set('rd', 'Rd*S_Vcyt');
model.variable('var6').set('tp', 'Tp*S_Vc');
model.variable('var6').set('vcmax', 'Vcmax*S_Vc');
model.variable('var6').set('o', 'O/(R*T)');
model.variable('var6').set('gamma', 'kH*Gamma/(R*T)');
model.variable('var6').set('kmc', 'kH*KmC/(R*T)');
model.variable('var6').set('kmo', 'KmO/(R*T)');
model.variable('var6').set('ci', 'Ci/(R*T)');
model.variable.create('var10');
model.variable('var10').model('comp1');
model.variable('var10').set('wc', 'c*vcmax/(c+kmc*(1+o/kmo))');
model.variable('var10').set('wj', 'c*j_/(4*c+8*gamma)');
model.variable('var10').set('wp', '3*tp/(max(eps,1-(gamma/c)))');
model.variable('var10').set('w', 'min(wc,min(wj,wp))');
model.variable.create('var9');
model.variable('var9').model('comp1');
model.variable('var9').set('W_1', 'intop1(w)*l_S');
model.variable('var9').set('F_1', 'intop1(w*gamma/c)*l_S');
model.variable('var9').set('F', '2*F_1*Vc_Vc1');
model.variable('var9').set('W', '2*W_1*Vc_Vc1');
model.variable.create('var11');
model.variable('var11').model('comp1');
model.variable('var11').set('R_MEM', '1/G_MEM');
model.variable('var11').set('G_WALL', 'DCO2_WALL/t_WALL');
model.variable('var11').set('R_WALL', '1/G_WALL');
model.variable('var11').set('R_WP', 'R_WALL+R_MEM');
model.variable('var11').set('G_WP', '1/R_WP');
model.variable.create('var16');
model.variable('var16').model('comp1');
model.variable('var16').set('cc', 'intop1(c)/intop1(1)');
model.variable('var16').set('cc_gas', 'cc/kH');
model.variable('var16').set('Cc', 'cc_gas*R*T');
model.variable('var16').set('An', 'W-F-Rd');
model.variable('var16').set('gm', 'An/(Ci-Cc)');

model.view.create('view2', 'geom1');
%
% Define a number of domains over which the model will calculate integrals.
model.cpl.create('intop1', 'Integration', 'geom1');
model.cpl.create('intop2', 'Integration', 'geom1');
model.cpl.create('intop3', 'Integration', 'geom1');
```

```
model.cpl.create('intop4', 'Integration', 'geom1');
model.cpl('intop1').selection.set([4]);
model.cpl('intop2').selection.set([1 2 3 5 6]);
model.cpl('intop3').selection.named('sel4');
model.cpl('intop4').selection.named('sel1');
%
% Define the partial differential equations and the boundary conditions
model.physics.create('tds', 'DilutedSpecies', 'geom1');
model.physics('tds').selection.named('uni4');
model.physics('tds').create('reac1', 'Reactions', 2);
model.physics('tds').feature('reac1').selection.named('sel2');
model.physics('tds').create('reac2', 'Reactions', 2);
model.physics('tds').feature('reac2').selection.named('sel4');
model.physics('tds').create('cdm2', 'ConvectionDiffusionMigration', 2);
model.physics('tds').feature('cdm2').selection.named('sel4');
model.physics('tds').create('cdm3', 'ConvectionDiffusionMigration', 2);
model.physics('tds').feature('cdm3').selection.named('uni1');
model.physics('tds').create('reac3', 'Reactions', 2);
model.physics('tds').feature('reac3').selection.named('sel2');
model.physics('tds').create('fl1', 'Fluxes', 1);
model.physics('tds').feature('fl1').selection.named('sel5');
model.physics('tds').create('tdb1', 'ThinDiffusionBarrier', 1);
model.physics('tds').feature('tdb1').selection.named('sel10');
%
model.variable('var15').label('Calculate dimensions computational
domains');
model.variable('var1').label('Calculate areas and volumes');
model.variable('var2').label('Photosynthesis parameters');
model.variable('var3').label('Diffusion coefficients');
model.variable('var4').label('Physical parameters');
model.variable('var5').label('Rates of electron transport');
model.variable('var6').label('Calculate volumetric parameters');
model.variable('var10').label('Calculate volumetric photosynthesis rate');
model.variable('var9').label('Calculate leaf photosynthesis');
model.variable('var11').label('Calculate mass transfer coefficient');
model.variable('var16').label('Calculate mesophyll conductance');
%
model.cpl('intop1').label('Integrate of stroma');
model.cpl('intop2').label('Integrate over cytosol');
%
model.physics('tds').label('Transport of C12');
model.physics('tds').prop('TransportMechanism').set('Convection', '0');
model.physics('tds').feature('cdm1').set('D_c', {'DCO2_CYT'; '0'; '0'; '0';
'DCO2_CYT'; '0'; '0'; '0'; 'DCO2_CYT'});
model.physics('tds').feature('init1').set('initc', 'ci*R*T/H');
model.physics('tds').feature('reac1').set('R_c', 'rd');
model.physics('tds').feature('reac1').label('Day respiration');
model.physics('tds').feature('reac2').set('R_c', '-w');
model.physics('tds').feature('reac2').label('CO2 assimilation');
model.physics('tds').feature('cdm2').set('D_c', {'DCO2_STR'; '0'; '0'; '0';
'DCO2_STR'; '0'; '0'; '0'; 'DCO2_STR'});
model.physics('tds').feature('cdm2').label('Diffusion in stroma');
model.physics('tds').feature('cdm3').set('D_c', {'DCO2_CYT'; '0'; '0'; '0';
'DCO2_CYT'; '0'; '0'; '0'; 'DCO2_CYT'});
model.physics('tds').feature('cdm3').label('Diffusion in cytosol');
model.physics('tds').feature('reac3').set('R_c',
'intop3(gamma*w/c)/intop4(1)');
model.physics('tds').feature('reac3').label('Photorespiration');
model.physics('tds').feature('fl1').set('FluxType',
'ExternalForcedConvection');
model.physics('tds').feature('fl1').set('species', '1');
```

```
model.physics('tds').feature('fl1').set('kc', 'G_WP');
model.physics('tds').feature('fl1').set('cb', 'ci*kH');
model.physics('tds').feature('fl1').label('Diffusion over cell wall and
plasma membrane');
model.physics('tds').feature('tdb1').set('ds', '1');
model.physics('tds').feature('tdb1').set('Ds', '0.5*3.5e-3');
model.physics('tds').feature('tdb1').label('Diffusion over chloroplast
enveloppe');
%
model.mesh('mesh1').run;
%%%%%%%%%%%%%%%%%%%%%%%%%%%%%%%%%%%%%%%%%%%%%%%%%%%%%%%%%%%%%%%%%%%%%%%%%%%
```

buildModelGaps.m

```
%%%%%%%%%%%%%%%%%%%%%%%%%%%%%%%%%%%%%%%%%%%%%%%%%%%%%%%%%%%%%%%%%%%%%%%%%%%
%
% Title:    buildModelGaps.m
%
% Authors:  dr. Herman N.C. Berghuijs
%           dr. Xinyou Yin
%           dr. Quang Tri Ho
%           dr. Moges A. Retta
%           prof. dr. Bart M. Nicolaï
%           prof. dr. Paul C. Struik
%
% Date:     March 11 2019
%
% For any questions regarding the code, please contact Herman Berghuijs
% (herman.berghuijs@slu.se / hermanberghuijs@hotmail.com)
%%%%%%%%%%%%%%%%%%%%%%%%%%%%%%%%%%%%%%%%%%%%%%%%%%%%%%%%%%%%%%%%%%%%%%%%%%%
%
% This file implements model equations, generates the geometry, defines
% the different subdomains within the geometry, and builds the mesh. In
% this M-file, it is assumed that CO2 produced by respiration and
% photorespiration is released in the cytosol gaps.
%
%%%%%%%%%%%%%%%%%%%%%%%%%%%%%%%%%%%%%%%%%%%%%%%%%%%%%%%%%%%%%%%%%%%%%%%%%%%
%
% Define and built the model geometry
model.geom('geom1').create('r1', 'Rectangle');
model.geom('geom1').feature('r1').setIndex('layer', 't_CYT', 0);
model.geom('geom1').feature('r1').set('layerbottom', false);
model.geom('geom1').feature('r1').set('size', {'t_STR+2*t_CYT'
'0.5*(h_GAP+h_STR)'});
model.geom('geom1').feature('r1').set('layername', {'Layer 1'});
model.geom('geom1').create('r2', 'Rectangle');
model.geom('geom1').feature('r2').set('layerbottom', false);
model.geom('geom1').feature('r2').set('size', {'t_STR'
'0.5*(h_STR+h_GAP)'});
model.geom('geom1').feature('r2').set('pos', {'t_CYT' '0'});
model.geom('geom1').create('r3', 'Rectangle');
model.geom('geom1').feature('r3').set('layer', {'t_CYT' 't_STR' 't_CYT'});
model.geom('geom1').feature('r3').set('layerbottom', false);
model.geom('geom1').feature('r3').set('size', {'t_STR+2*t_CYT'
'0.5*h_GAP'});
model.geom('geom1').feature('r3').set('pos', {'0' '0'});
model.geom('geom1').feature('r3').set('layername', {'Layer 1' 'Layer 1' ''
''});
model.geom('geom1').run;

model.selection.create('sel1', 'Explicit');
model.selection('sel1').set([5 6]);
model.selection.create('sel2', 'Explicit');
model.selection('sel2').set([1 2]);
model.selection.create('sel3', 'Explicit');
model.selection('sel3').set([3]);
model.selection.create('sel4', 'Explicit');
model.selection('sel4').set([4]);
model.selection.create('sel5', 'Explicit');
model.selection('sel5').geom('geom1', 1);
model.selection('sel5').set([1 3]);
```

```
model.selection.create('sel6', 'Explicit');
model.selection('sel6').geom('geom1', 1);
model.selection('sel6').set([16 17]);
model.selection.create('uni1', 'Union');
model.selection.create('uni2', 'Union');
model.selection.create('uni3', 'Union');
model.selection.create('uni4', 'Union');
model.selection.create('sel8', 'Explicit');
model.selection('sel8').geom('geom1', 1);
model.selection.create('uni5', 'Union');
model.selection.create('sel10', 'Explicit');
model.selection('sel10').geom('geom1', 1);
model.selection('sel10').set([8 9 13]);
model.selection('sel1').label('Inner cytosol');
model.selection('sel1').label('Explicit 1');
model.selection('sel2').label('Outer cytosol');
model.selection('sel2').label('Explicit 2');
model.selection('sel3').label('Cytosol gap');
model.selection('sel3').label('Explicit 3');
model.selection('sel4').label('Stroma');
model.selection('sel4').label('Explicit 4');
model.selection('sel5').label('Cell wall and plasma membrane');
model.selection('sel5').label('Explicit 5');
model.selection('sel6').label('Tonoplast');
model.selection('sel6').label('Explicit 6');
model.selection('uni1').set('input', {'sel1' 'sel2' 'sel3'});
model.selection('uni1').label('Cytosol');
model.selection('uni2').set('input', {'sel1' 'sel4'});
model.selection('uni2').label('Stroma and inner cytosol');
model.selection('uni3').set('input', {'sel2' 'sel4'});
model.selection('uni3').label('Stroma and outer cytosol');
model.selection('uni4').set('input', {'sel4' 'uni1'});
model.selection('uni4').label('Liquid phase');
model.selection('sel8').label('Stomata');
model.selection('sel8').label('Explicit 8');
model.selection('uni5').set('input', {'sel1' 'sel2'});
model.selection('uni5').label('Inner and outer cytosol');
model.selection('sel10').label('Chloroplast envelope');
model.selection('sel10').label('Explicit 10');

model.variable.create('var15');
model.variable('var15').model('comp1');
model.variable('var15').set('h_STR', 'ratio*t_STR');
model.variable('var15').set('h_GAP', 'ratio*t_STR*((Sc_Sm^-1)-1)');
model.variable.create('var1');
model.variable('var1').model('comp1');
model.variable('var1').set('S_Vc', '(1/t_STR)*(Sm_S^-1)*(Sc_Sm^-1)');
model.variable('var1').set('S_Vcyt1', '(1/t_CYT)*(Sm_S^-1)');
model.variable('var1').set('S_Vcyt12', '(1/(2*t_CYT))*(Sm_S^-1)');
model.variable('var1').set('S_Vcyt', 'S_Vgap');
model.variable('var1').set('Vc_Vc1',
'((2*t_CYT+t_STR)*Sm_S*Sc_Sm)/(ratio*t_STR)');
model.variable('var1').set('l_S', '1/(2*t_CYT+t_STR)');
model.variable('var1').set('S_Vgap', '(1/t_STR)*(Sm_S*(1-Sc_Sm))^-1');
model.variable.create('var2');
model.variable('var2').model('comp1');
model.variable('var2').set('Gamma', '0.5*O/ScO');
model.variable.create('var3');
model.variable('var3').model('comp1');
model.variable('var3').set('p_eff_WALL', '0.2');
model.variable('var3').set('zeta_STR', '0.5');
```

```
model.variable('var3').set('zeta_CYT', '0.5');
model.variable('var3').set('G_MEM', '3.5e-3[m][s^-1]');
model.variable('var3').set('DCO2_WATER', '1.79e-9[m^2][s^-1]');
model.variable('var3').set('DHCO3_WATER', '0.52*DCO2_WATER');
model.variable('var3').set('DCO2_WALL', 'p_eff_WALL*DCO2_WATER');
model.variable('var3').set('DCO2_CYT', 'zeta_CYT*DCO2_WATER');
model.variable('var3').set('DCO2_STR', 'zeta_STR*DCO2_WATER');
model.variable.create('var4');
model.variable('var4').model('comp1');
model.variable('var4').set('R', '8.314[Pa][m^3][mol^-1][K^-1]');
model.variable('var4').set('H', '2941[Pa][m^3][mol^-1]');
model.variable('var4').set('kH', 'R*T/H');
model.variable('var4').set('T', '298.15[K]');
model.variable.create('var5');
model.variable('var5').model('comp1');
model.variable('var5').set('j_', 'J*S_Vc');
model.variable('var5').set('Iinc', 'Iinc0');
model.variable.create('var6');
model.variable('var6').model('comp1');
model.variable('var6').set('rd', 'Rd*S_Vcyt');
model.variable('var6').set('tp', 'Tp*S_Vc');
model.variable('var6').set('vcmax', 'Vcmax*S_Vc');
model.variable('var6').set('o', 'O/(R*T)');
model.variable('var6').set('gamma', 'kH*Gamma/(R*T)');
model.variable('var6').set('kmc', 'kH*KmC/(R*T)');
model.variable('var6').set('kmo', 'KmO/(R*T)');
model.variable('var6').set('ci', 'Ci/(R*T)');
model.variable.create('var10');
model.variable('var10').model('comp1');
model.variable('var10').set('wc', 'c*vcmax/(c+kmc*(1+o/kmo))');
model.variable('var10').set('wj', 'c*j_/(4*c+8*gamma)');
model.variable('var10').set('wp', '3*tp/(max(eps,1-(gamma/c)))');
model.variable('var10').set('w', 'min(wc,min(wj,wp))');
model.variable.create('var9');
model.variable('var9').model('comp1');
model.variable('var9').set('W_1', 'intop1(w)*l_S');
model.variable('var9').set('F_1', 'intop1(w*gamma/c)*l_S');
model.variable('var9').set('F', '2*F_1*Vc_Vc1');
model.variable('var9').set('W', '2*W_1*Vc_Vc1');
model.variable.create('var11');
model.variable('var11').model('comp1');
model.variable('var11').set('R_MEM', '1/G_MEM');
model.variable('var11').set('G_WALL', 'DCO2_WALL/t_WALL');
model.variable('var11').set('R_WALL', '1/G_WALL');
model.variable('var11').set('R_WP', 'R_WALL+R_MEM');
model.variable('var11').set('G_WP', '1/R_WP');
model.variable.create('var16');
model.variable('var16').model('comp1');
model.variable('var16').set('cc', 'intop1(c)/intop1(1)');
model.variable('var16').set('cc_gas', 'cc/kH');
model.variable('var16').set('Cc', 'cc_gas*R*T');
model.variable('var16').set('An', 'W-F-Rd');
model.variable('var16').set('gm', 'An/(Ci-Cc)');

model.view('view1').tag('view2');

model.cpl.create('intop1', 'Integration', 'geom1');
model.cpl.create('intop2', 'Integration', 'geom1');
model.cpl.create('intop3', 'Integration', 'geom1');
model.cpl.create('intop4', 'Integration', 'geom1');
model.cpl('intop1').selection.set([4]);
```

```
model.cpl('intop2').selection.set([1 2 3 5 6]);
model.cpl('intop3').selection.named('sel4');
model.cpl('intop4').selection.named('sel3');

model.physics.create('tds', 'DilutedSpecies', 'geom1');
model.physics('tds').selection.named('uni4');
model.physics('tds').create('reac1', 'Reactions', 2);
model.physics('tds').feature('reac1').selection.named('sel3');
model.physics('tds').create('reac2', 'Reactions', 2);
model.physics('tds').feature('reac2').selection.named('sel4');
model.physics('tds').create('cdm2', 'ConvectionDiffusionMigration', 2);
model.physics('tds').feature('cdm2').selection.named('sel4');
model.physics('tds').create('cdm3', 'ConvectionDiffusionMigration', 2);
model.physics('tds').feature('cdm3').selection.named('uni1');
model.physics('tds').create('reac3', 'Reactions', 2);
model.physics('tds').feature('reac3').selection.named('sel3');
model.physics('tds').create('fl1', 'Fluxes', 1);
model.physics('tds').feature('fl1').selection.named('sel5');
model.physics('tds').create('tdb1', 'ThinDiffusionBarrier', 1);
model.physics('tds').feature('tdb1').selection.named('sel10');

model.variable('var15').label('Calculate dimensions computational
domains');
model.variable('var1').label('Calculate areas and volumes');
model.variable('var2').label('Photosynthesis parameters');
model.variable('var3').label('Diffusion coefficients');
model.variable('var4').label('Physical parameters');
model.variable('var5').label('Rates of electron transport');
model.variable('var6').label('Calculate volumetric parameters');
model.variable('var10').label('Calculate volumetric photosynthesis rate');
model.variable('var9').label('Calculate leaf photosynthesis');
model.variable('var11').label('Calculate mass transfer coefficient');
model.variable('var16').label('Calculate mesophyll conductance');

model.view('view2').label('View 2');
model.view('view2').axis.set('abstractviewxscale', '8.616719782139626E-9');
model.view('view2').axis.set('ymin', '-6.72887608743622E-7');
model.view('view2').axis.set('xmax', '4.540368991001742E-6');
model.view('view2').axis.set('abstractviewyscale', '8.616719782139626E-9');
model.view('view2').axis.set('abstractviewbratio', '-
0.050000011920928955');
model.view('view2').axis.set('abstractviewtratio', '0.050000011920928955');
model.view('view2').axis.set('abstractviewrratio', '0.15368618071079254');
model.view('view2').axis.set('xmin', '-1.5085685163285234E-6');
model.view('view2').axis.set('abstractviewlratio', '-0.15368613600730896');
model.view('view2').axis.set('ymax', '4.135242306801956E-6');

model.cpl('intop1').label('Integrate of stroma');
model.cpl('intop2').label('Integrate over cytosol');

model.physics('tds').label('Transport of C12');
model.physics('tds').prop('TransportMechanism').set('Convection', '0');
model.physics('tds').feature('cdm1').set('D_c', {'DCO2_CYT'; '0'; '0'; '0';
'DCO2_CYT'; '0'; '0'; '0'; 'DCO2_CYT'});
model.physics('tds').feature('init1').set('initc', 'ci*R*T/H');
model.physics('tds').feature('reac1').set('R_c', 'rd');
model.physics('tds').feature('reac1').label('Day respiration');
model.physics('tds').feature('reac2').set('R_c', '-w');
model.physics('tds').feature('reac2').label('CO2 assimilation');
model.physics('tds').feature('cdm2').set('D_c', {'DCO2_STR'; '0'; '0'; '0';
'DCO2_STR'; '0'; '0'; '0'; 'DCO2_STR'});
```

```
model.physics('tds').feature('cdm2').label('Diffusion in stroma');
model.physics('tds').feature('cdm3').set('D_c', {'DCO2_CYT'; '0'; '0'; '0';
'DCO2_CYT'; '0'; '0'; '0'; 'DCO2_CYT'});
model.physics('tds').feature('cdm3').label('Diffusion in cytosol');
model.physics('tds').feature('reac3').set('R_c',
'intop3(gamma*w/c)/intop4(1)');
model.physics('tds').feature('reac3').label('Photorespiration');
model.physics('tds').feature('fl1').set('FluxType',
'ExternalForcedConvection');
model.physics('tds').feature('fl1').set('species', '1');
model.physics('tds').feature('fl1').set('kc', 'G_WP');
model.physics('tds').feature('fl1').set('cb', 'ci*kH');
model.physics('tds').feature('fl1').label('Diffusion over cell wall and
plasma membrane');
model.physics('tds').feature('tdb1').set('ds', '1');
model.physics('tds').feature('tdb1').set('Ds', '0.5*3.5e-3');
model.physics('tds').feature('tdb1').label('Diffusion over chloroplast
enveloppe');
%
model.mesh('mesh1').run;
%%%%%%%%%%%%%%%%%%%%%%%%%%%%%%%%%%%%%%%%%%%%%%%%%%%%%%%%%%%%%%%%%%%%%%%%%%%%
%%%%%%%%%%%%%%%%%%%%
```

CalculateResidualRd.m

```
%%%%%%%%%%%%%%%%%%%%%%%%%%%%%%%%%%%%%%%%%%%%%%%%%%%%%%%%%%%%%%%%%%%%%%%%%%
%
% Title:    calculateResidualRd.m
%
% Authors:  dr. Herman N.C. Berghuijs
%           dr. Xinyou Yin
%           dr. Quang Tri Ho
%           dr. Moges A. Retta
%           prof. dr. Bart M. Nicolaï
%           prof. dr. Paul C. Struik
%
% Date:     March 11 2019
%
% For any questions regarding the code, please contact Herman Berghuijs
% (herman.berghuijs@slu.se / hermanberghuijs@hotmail.com)
%%%%%%%%%%%%%%%%%%%%%%%%%%%%%%%%%%%%%%%%%%%%%%%%%%%%%%%%%%%%%%%%%%%%%%%%%%
%
% The aim of the function in this M-file is to calculate the sum of the
% residuals between the measured net CO2 assimilation rates that were used
% to estimate Rd and the simulated values.
%
%%%%%%%%%%%%%%%%%%%%%%%%%%%%%%%%%%%%%%%%%%%%%%%%%%%%%%%%%%%%%%%%%%%%%%%%%%
%
function residuals=calculateResidualRd(Rd)
%
    dataRd;
    An_data=An_dataRd;
    Iinc_data=Iinc_dataRd;
    Ci_data=Ci_dataRd;
    J_data=J_dataRd;
%
    display(['Rd=', num2str(Rd)]);
    % Declare vector to store modelled rates of net CO2 assimilation
    An_model=zeros(1,length(Iinc_data));
%
    % Load model
    model=mphload([pwd,'\model2.mph']);
%
    for i=1:length(Iinc_data)
            model.param.set('Iinc0', [num2str(Iinc_data(i)),'[umol][m^-
2][s^-1]']);
            model.param.set('Ci', [num2str(Ci_data(i)),'[Pa]']);
            model.param.set('Rd', [num2str(Rd),'[umol][m^-2][s^-1]']);
            model.variable('var5').set('J', [num2str(J_data(i)),'[umol][m^-
2][s^-1]']);
%
            solveModel;
            W=mphglobal(model,'W');
            F=mphglobal(model,'F');
            Rd_est=mphglobal(model,'Rd');
            An_model(i)=1e6*(W-F-Rd_est);
    end
%
    residuals=(An_model'-An_data').^2;
end
%%%%%%%%%%%%%%%%%%%%%%%%%%%%%%%%%%%%%%%%%%%%%%%%%%%%%%%%%%%%%%%%%%%%%%%%%%
```

DataRd

```
%%%%%%%%%%%%%%%%%%%%%%%%%%%%%%%%%%%%%%%%%%%%%%%%%%%%%%%%%%%%%%%%%%%%%%%%%%
%
% Title:    dataRd.m
%
% Authors:  dr. Herman N.C. Berghuijs
%           dr. Xinyou Yin
%           dr. Quang Tri Ho
%           dr. Moges A. Retta
%           prof. dr. Bart M. Nicolaï
%           prof. dr. Paul C. Struik
%
% Date:     March 11 2019
%
% For any questions regarding the code, please contact Herman Berghuijs
% (herman.berghuijs@slu.se / hermanberghuijs@hotmail.com)
%%%%%%%%%%%%%%%%%%%%%%%%%%%%%%%%%%%%%%%%%%%%%%%%%%%%%%%%%%%%%%%%%%%%%%%%%%
% The aim of this code is to load the light response curve measurements
% that are used to estimate Rd with the reaction diffusion model.
%%%%%%%%%%%%%%%%%%%%%%%%%%%%%%%%%%%%%%%%%%%%%%%%%%%%%%%%%%%%%%%%%%%%%%%%%%
%
% Observed net CO2 assimilation rate (umol m-2 s-1)
An_dataRd=[5.856395852,2.904632356,-0.299575675,-1.279395273];
%
% Observed ambient CO2 partial pressure (Pa)
Ca_dataRd=[40.04726334,40.02993698,40.03736954,40.0433342];
%
% Observed intercellular CO2 partial pressure (Pa)
Ci_dataRd =[34.4021,36.1239,39.1888,40.4919];
%
% Observed irradiances (umol m-2 s-1)
Iinc_dataRd=[149.3584557,100.3891029,51.48970413,23.60690546];
%
% Calculated rates of electron transport (J = s * PSII), where J is the
% rate of electron transport and s is a lumped calibration faction.
% (umol m-2 s-1)
J_dataRd=[51.96434159,36.29423493,19.29827995,9.031788168];
%
% Calculate stomatal conductance
gs_dataRd=An_dataRd./(Ca_dataRd-Ci_dataRd);

save An_dataRd An_dataRd
save gs_dataRd gs_dataRd
save Ca_dataRd Ca_dataRd
save Ci_dataRd Ci_dataRd
save Iinc_dataRd Iinc_dataRd
%%%%%%%%%%%%%%%%%%%%%%%%%%%%%%%%%%%%%%%%%%%%%%%%%%%%%%%%%%%%%%%%%%%%%%%%%%
```

CalculateResidualVcmax.m

```
%%%%%%%%%%%%%%%%%%%%%%%%%%%%%%%%%%%%%%%%%%%%%%%%%%%%%%%%%%%%%%%%%%%%%%%%%
%
% Title:    calculateResidualVcmax.m
%
% Authors:  dr. Herman N.C. Berghuijs
%           dr. Xinyou Yin
%           dr. Quang Tri Ho
%           dr. Moges A. Retta
%           prof. dr. Bart M. Nicolaï
%           prof. dr. Paul C. Struik
%
% Date:     March 11 2019
%
% For any questions regarding the code, please contact Herman Berghuijs
% (herman.berghuijs@slu.se / hermanberghuijs@hotmail.com)
%%%%%%%%%%%%%%%%%%%%%%%%%%%%%%%%%%%%%%%%%%%%%%%%%%%%%%%%%%%%%%%%%%%%%%%%%
%
% The aim of the function in this M-file is to calculate the sum of the
% residuals between the measured net CO2 assimilation rates that were used
% to estimate Vcmax and the simulated values.
%
%%%%%%%%%%%%%%%%%%%%%%%%%%%%%%%%%%%%%%%%%%%%%%%%%%%%%%%%%%%%%%%%%%%%%%%%%
%
function residuals=calculateResidualVcmax(Vcmax)
%
    dataVcmax;
    An_data=An_dataVcmax;
    Iinc_data=Iinc_dataVcmax;
    Ci_data=Ci_dataVcmax;
    J_data=J_dataVcmax;
%
    display(['Vcmax=', num2str(Vcmax)]);
    % Declare vector to store modelled rates of net CO2 assimilation
    An_model=zeros(1,length(Iinc_data));
%
    % Load model
    model=mphload([pwd,'\model2.mph']);
%
    for i=1:length(Iinc_data)
            model.param.set('Iinc0', [num2str(Iinc_data(i)),'[umol][m^-
2][s^-1]']);
            model.param.set('Ci', [num2str(Ci_data(i)),'[Pa]']);
            model.param.set('Vcmax', [num2str(Vcmax),'[umol][m^-2][s^-
1]']);
            model.variable('var5').set('J', [num2str(J_data(i)),'[umol][m^-
2][s^-1]']);
%
            solveModel;
            W=mphglobal(model,'W');
            F=mphglobal(model,'F');
            Rd_est=mphglobal(model,'Rd');
            An_model(i)=1e6*(W-F-Rd_est);
    end
 %
    residuals=An_model'-An_data';
  %
```

```
end

%%%%%%%%%%%%%%%%%%%%%%%%%%%%%%%%%%%%%%%%%%%%%%%%%%%%%%%%%%%%%%%%%%%%%%%%%%
```

DataVcmax.m

```
%%%%%%%%%%%%%%%%%%%%%%%%%%%%%%%%%%%%%%%%%%%%%%%%%%%%%%%%%%%%%%%%%%%%%%%%%%%
%
% Title:    dataVcmax.m
%
% Authors:  dr. Herman N.C. Berghuijs
%           dr. Xinyou Yin
%           dr. Quang Tri Ho
%           dr. Moges A. Retta
%           prof. dr. Bart M. Nicolaï
%           prof. dr. Paul C. Struik
%
% Date:     March 11 2019
%
% For any questions regarding the code, please contact Herman Berghuijs
% (herman.berghuijs@slu.se / hermanberghuijs@hotmail.com)
%%%%%%%%%%%%%%%%%%%%%%%%%%%%%%%%%%%%%%%%%%%%%%%%%%%%%%%%%%%%%%%%%%%%%%%%%%%
% The aim of this code is to load the light response curve measurements
% that are used to estimate Vcmax with the reaction diffusion model.
%%%%%%%%%%%%%%%%%%%%%%%%%%%%%%%%%%%%%%%%%%%%%%%%%%%%%%%%%%%%%%%%%%%%%%%%%%%
%
% Observed ambient CO2 partial pressure (Pa)
Ca_dataVcmax =[29.93387146,20.022583,9.940552521,5.080346298];
%
% Observed intercellular CO2 partial pressure (Pa)
Ci_dataVcmax = [18.98789412,13.17666079,7.961297167,5.39349546];
%
% Observed net CO2 assimilation rate (umol m-2 s-1)
An_dataVcmax = [17.89843384,11.83933345,3.667620745,-0.971040286];
%
% Observed irradiances (umol m-2 s-1)
Iinc_dataVcmax=[1499.87265,1499.819733,1499.69812     1499.603577];
%
% Calculated rates of electron transport (J = s * PSII), where J is the
% rate of electron transport and s is a lumped calibration faction.
% (umol m-2 s-1)
J_dataVcmax=[192.7053082,173.9048405,147.0389258,126.1549297];
%
% Calculate intercellular CO2 partial pressure
gs_dataVcmax = An_dataVcmax/(Ca_dataVcmax-Ci_dataVcmax);
%
save An_dataVcmax An_dataVcmax
save gs_dataVcmax gs_dataVcmax
save Ca_dataVcmax Ca_dataVcmax
save Ci_dataVcmax Ci_dataVcmax
save Iinc_dataVcmax Iinc_dataVcmax
save J_dataVcmax J_dataVcmax
%%%%%%%%%%%%%%%%%%%%%%%%%%%%%%%%%%%%%%%%%%%%%%%%%%%%%%%%%%%%%%%%%%%%%%%%%%%%
```

```
%%%%%%%%%%%%%%%%%%%%%%%%%%%%%%%%%%%%%%%%%%%%%%%%%%%%%%%%%%%%%%%%%%%%%%%%%
%
% Title:    solveModel.m
%
% Authors:  dr. Herman N.C. Berghuijs
%           dr. Xinyou Yin
%           dr. Quang Tri Ho
%           dr. Moges A. Retta
%           prof. dr. Bart M. Nicolaï
%           prof. dr. Paul C. Struik
%
% Date:     March 11 2019
%
% For any questions regarding the code, please contact Herman Berghuijs
% (herman.berghuijs@slu.se / hermanberghuijs@hotmail.com)
%%%%%%%%%%%%%%%%%%%%%%%%%%%%%%%%%%%%%%%%%%%%%%%%%%%%%%%%%%%%%%%%%%%%%%%%%
% The aim of this code is to numerically solve the model
%%%%%%%%%%%%%%%%%%%%%%%%%%%%%%%%%%%%%%%%%%%%%%%%%%%%%%%%%%%%%%%%%%%%%%%%%
%
model.sol('sol1').runAll;
%%%%%%%%%%%%%%%%%%%%%%%%%%%%%%%%%%%%%%%%%%%%%%%%%%%%%%%%%%%%%%%%%%%%%%%%%
```

References

Berghuijs HNC, Yin X, Ho QT, Retta MA, Verboven P, Nicolaï, B. M., Struik PC. 2017. Localization of (photo) respiration and CO2 re-assimilation in tomato leaves investigated with a reaction-diffusion model. *Plos One* **12**(9).

Berghuijs HNC, Yin X, Ho QT, van der Putten PEL, Verboven P, Retta MA, Nicolaï BM, Struik PC. 2015. Modelling the relationship between CO_2 assimilation and leaf anatomical properties in tomato leaves. *Plant Science* **238**: 297-311.

Burnham KP, Anderson DR. 2004. Multimodel interference - understanding AIC and BIC in model selection. *Sociological Methods & Research* **33**: 261-304.

Ho QT, Berghuijs HNC, Watte R, Verboven P, Herremans E, Yin XY, Retta MA, Aernouts B, Saeys W, Helfen L, et al. 2016. Three-dimensional microscale modelling of CO_2 transport and light propagation in tomato leaves enlightens photosynthesis. *Plant Cell and Environment* **39**(1): 50-61.

Tholen D, Zhu XG. 2011. The mechanistic basis of internal conductance: A theoretical analysis of mesophyll cell photosynthesis and CO_2 diffusion. *Plant Physiology* **156**(1): 90-105.

Supporting Tables and Figure legends

Figure S1: Measured versus simulated CO_2 response curves under photorespiratory and non-photorespiratory conditions.

Figure S2: Measured versus simulated light response curves under photorespiratory conditons and non-photorespiratory conditions.

Figure S3: Day respiration rate estimates obtained by the reaction diffusion model versus estimates obtained by the Yin method.

Figure S4 : Schematic overview of the flow of the program that was used to estimate parameter values with the reaction diffusion model.

Table S1: Estimates of the lumped calibration factors and the day respiration rates obtained by various methods using data from Berghuijs *et al*. (2015).

Table S2: Estimates of the lumped calibration factors and the day respiration rates obtained by various methods using data from Ho *et al*. (2016).

Table S3: Estimates of the maximum RuBP carboxylation rate by Rubisco and the triose phosphate utilization rates obtained by the reaction-diffusion model for different scenarios of (photo)respired CO_2 release.

Table S4: Akaike's information criteria for different combinations of leaf age, cultivar, photorespiratory conditions, and scenarios for the release of (photo)respired CO_2 using the reaction-diffusion model and data from Berghuijs *et al*. (2015)

Table S5: Akaike's information criteria for different combinations of leaf age, cultivar, photorespiratory conditions, and scenarios for the release of (photo)respired CO_2 using the reaction-diffusion model and data from Ho *et al*. (2016)

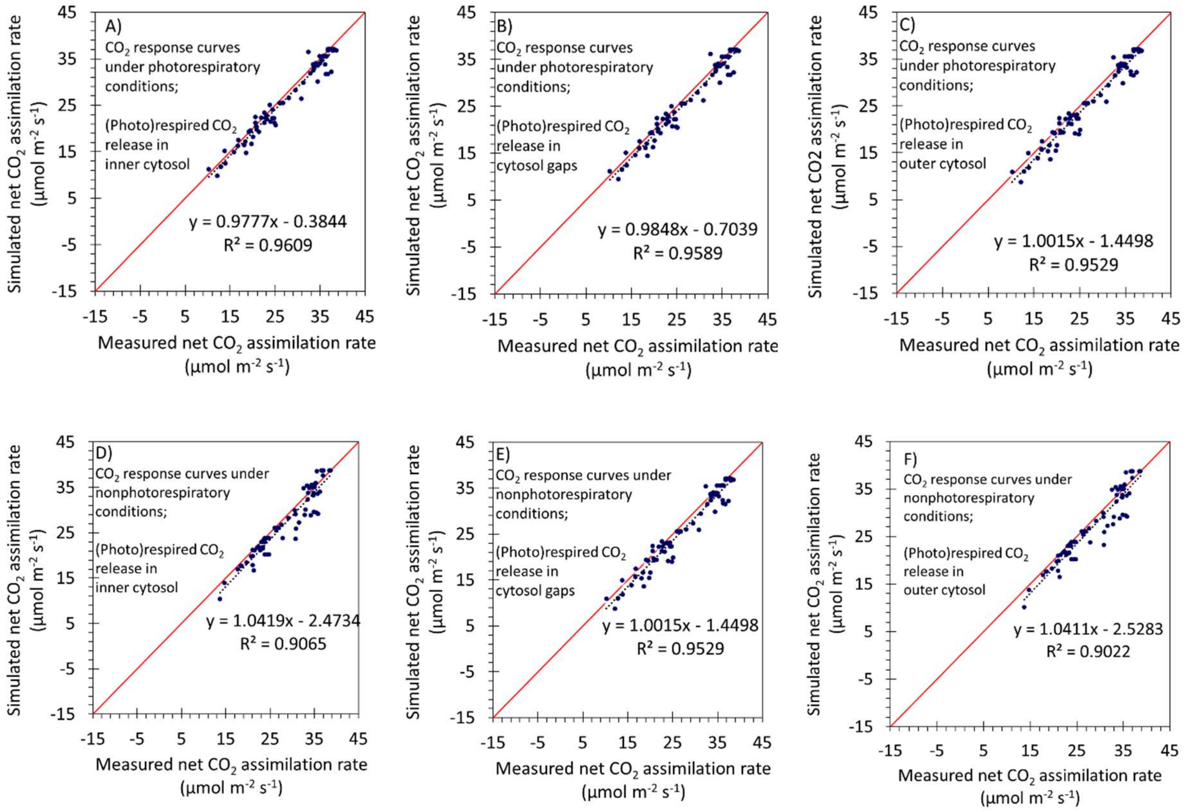

Fig. S1: Measured versus simulated CO_2 response curves under photorespiratory conditions (A-C; saturating light and 21 kPa O_2) an non-photorespiratory conditions (D-F; saturating light, 2 kPA O_2). The solid line is the 1:1 line. The dotted line represents a fitted linear regression line. The equation describes this regression line. (Photo)respired CO_2 release is assumed to take place in the inner cytosol (A,D), cytosol gaps (B,E) or outer cytosol (C,F).

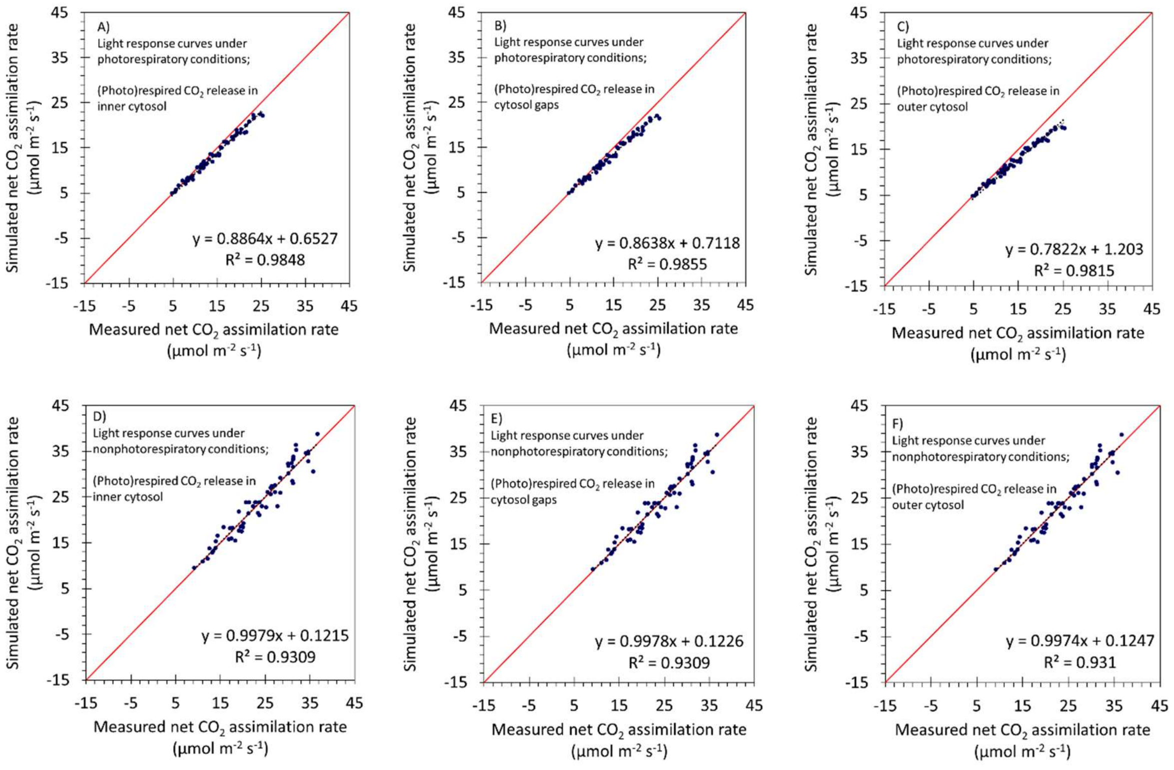

Fig. S2: Measured versus simulated light response curves under photorespiratory conditons (A-C; ambient CO_2 and 21 kPa O_2) and non-photorespiratory conditions (D-F; 100 Pa CO_2 and 2 kPa O_2). The solid line is the 1-1 line. The dotted line represents a fitted linear regression line. The equation describes this regression line. (Photo)respired CO_2 release is assumed to place in the inner cytosol (A,D), cytosol gaps (B,E) or outer cytosol (C,F).

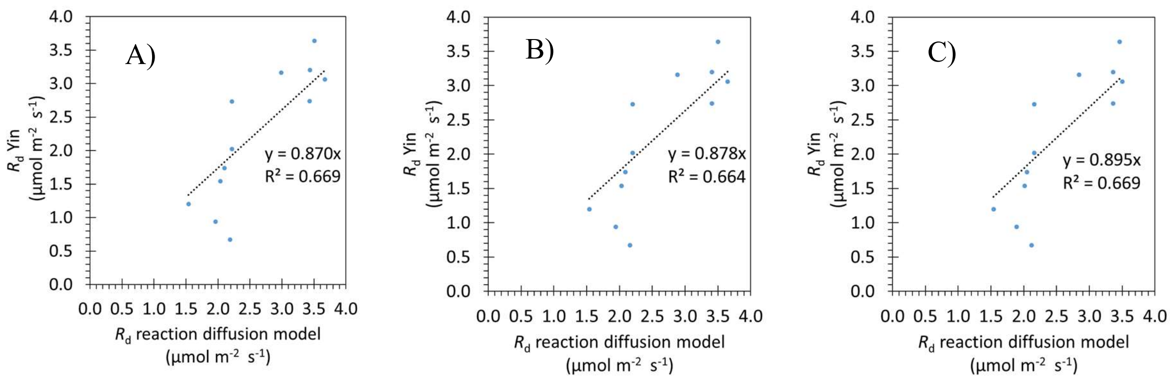

Fig. S3: R_d estimated by the reaction diffusion model versus R_d estimated by the Yin method. It is either assumed that (photo)respired CO_2 is released in the inner cytosol (A), the cytosol gaps (B) or the outer cytosol (C).

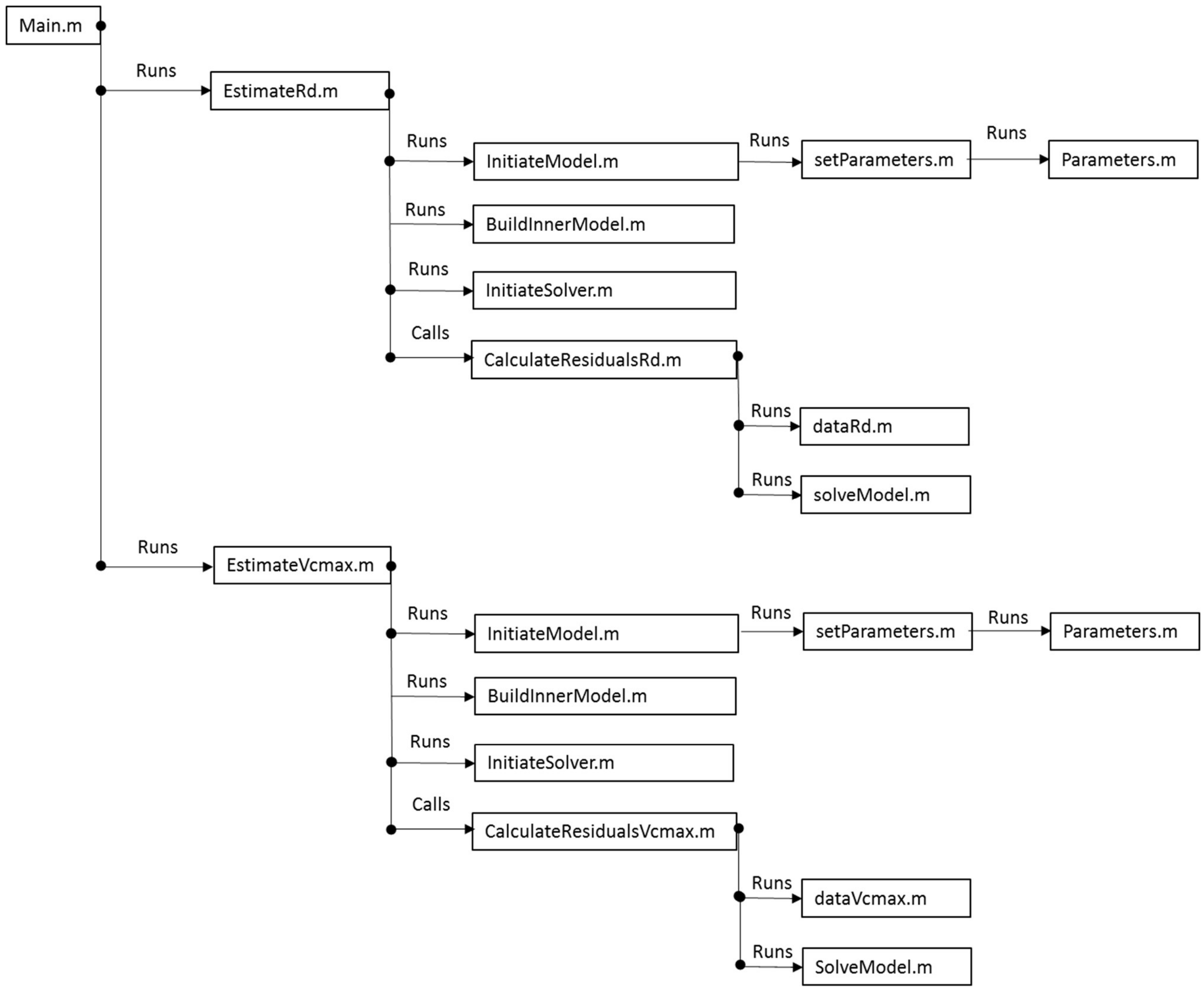

Fig. S4 : Schematic overview of the flow of the program. The rectangles represent M-files. An arrow from one file to another indicates that the first file runs the latter ones or, in case of CalculateResidualsRd.m and CalculateResidualsVcmax.m, calls a function inside these files.

Table S1: Estimates of the lumped calibration factor s and R_d, either estimated by the Kok (1948) method, the Yin *et al*. (2009) method or by the reaction-diffusion model for three locations of (photo)respiration. Data from Berghuijs *et al.* (2015) for three cultivars and two leaf ages were used for estimation. Estimates were made both for photorespiratory (PR, i.e., $C_a = 40$ Pa, $O = 21$ kPa) and nonphotorespiratory (NPR, i.e., $C_a = 100$ Pa, $O = 2$ kPa) conditions

Cultivar	Leaf age (d)	s	Condition	R_d (μmol m^{-2} s^{-1}) [4]				
				Kok	Yin	Reaction-diffusion model		
						Inner[1]	Gaps[2]	Outer[3]
Admiro	15	0.53±0.03	PR	2.95±0.33	3.20±0.34	3.44±0.36	3.41±0.36	3.36±0.36
			NPR	1.71±0.43	2.12±0.53	2.04±0.61	2.04±0.61	2.04±0.61
	25	0.52±0.03	PR	2.54±0.34	2.74±0.37	3.43±0.36	3.41±0.36	3.36±0.36
			NPR	1.26±0.43	1.64±0.46	1.74±0.27	1.74±0.27	1.74±0.27
Doloress	15	0.51±0.03	PR	2.87±0.22	3.06±0.23	3.67±0.32	3.65±0.32	3.60±0.33
			NPR	1.92±0.51	2.32±0.52	2.24±0.33	2.24±0.33	2.24±0.33
	25	0.41±0.03	PR	3.44±0.45	3.64±0.49	3.51±0.31	3.50±0.32	3.46±0.33
			NPR	0.77±0.34	1.08±0.41	1.01±0.10	1.00±0.10	1.01±0.10
Growdena	15	0.46±0.04	PR	2.91±0.46	3.16±0.45	2.90±0.39	2.88±0.40	2.84±0.41
			NPR	0.85±0.61	1.23±0.65	1.15±0.66	1.15±0.66	1.15±0.66
	25	0.47±0.03	PR	2.57±0.27	2.73±0.34	2.22±0.41	2.20±0.42	2.16±0.42
			NPR	0.79±0.33	1.17±0.47	1.15±0.66	1.15±0.66	1.15±0.66

[1] Inner: This column contains the values of R_d estimated by the reaction-diffusion model for the scenario that assumed release of CO_2 from (photo)respiration in the inner cytosol.
[2] Gaps: This column contains the values of R_d estimated by the reaction-diffusion model for the scenario that assumed release of CO_2 from (photo)respiration in the cytosol gaps between chloroplasts.
[3] Outer: This column contains the values of R_d estimated by the reaction-diffusion model for the scenario that assumed release of CO_2 from (photo)respiration in the outer cytosol.
[4] Estimated value of R_d ± standard deviation.

Table S2: Estimates of the lumped calibration factor s and R_d, either determined by the Kok (1948) method, the Yin *et al*. (2009) method or by the reaction-diffusion model (Berghuijs *et al.*, 2017). Data from Ho *et al.* (2016) were used for estimation. Estimates were done under both photorespiratory (PR, i.e., $C_a = 38$ Pa, $O = 21$ kPa) and nonphotorespiratory (NPR, i.e., $C_a = 100$ Pa, $O = 2$ kPa) conditions

Cultivar	Leaf type	s	Condition	R_d (µmol m^{-2} s^{-1})[4]				
				Kok	Yin	Reaction-diffusion model		
						Inner[1]	Gap[2]	Outer[3]
Admiro	Upper	0.52±0.03	PR	1.35±0.28	1.54±0.28	2.04±0.28	2.03±0.28	2.01±0.27
			NPR	2.05±0.42	2.18±0.44	1.99±0.49	1.99±0.49	1.99±0.49
	Lower	0.41±0.03	PR	0.98±0.30	1.20±0.29	1.54±0.27	1.54±0.26	1.54±0.26
			NPR	0.53±0.48	0.84±0.41	0.62±0.32	0.62±0.32	0.62±0.32
Doloress	Upper	0.49±0.03	PR	1.58±0.24	1.74±0.22	2.10±0.19	2.09±0.18	2.05±0.18
			NPR	1.64±0.45	1.83±0.45	1.56±0.36	1.56±0.36	1.56±0.36
	Lower	0.46±0.03	PR	0.77±0.29	0.94±0.29	1.96±0.07	1.94±0.09	1.89±0.12
			NPR	0.87±0.47	1.26±0.35	1.44±0.30	1.44±0.30	1.44±0.30
Growdena	Upper	0.50±0.03	PR	1.81±0.30	2.02±0.26	2.22±0.11	2.20±0.09	2.16±0.07
			NPR	1.68±0.30	1.74±0.32	1.42±0.25	1.42±0.25	1.42±0.25
	Lower	0.48±0.02	PR	0.46±0.41	0.67±0.38	2.19±0.07	2.16±0.09	2.12±0.12
			NPR	0.66±0.76	1.45±0.40	1.33±0.26	1.33±0.26	1.33±0.26

[1] Inner: This column contains the values of R_d estimated by the reaction-diffusion model for the scenario that assumed release of CO_2 from (photo)respiration in the inner cytosol.
[2] Gaps: This column contains the values of R_d estimated by the reaction diffusion model for the scenario that assumed release of CO_2 from (photo)respiration in the cytosol gaps between chloroplasts.
[3] Outer: This column contains the values of R_d estimated by the reaction diffusion model for the scenario that assumed release of CO_2 from (photo)respiration in the outer cytosol.
[4] Estimated value of R_d ± standard deviation.

**Table S3:** Values for T_p and V_{cmax} estimates for different scenarios of the location of (photo)respiratory CO_2 release								
			T_p (µmol m^{-2} s^{-1})			V_{cmax} (µmol m^{-2} s^{-1})		
			Inner[1]	Gaps[2]	Outer[3]	Inner[1]	Gaps[2]	Outer[3]
Data of Berghuijs *et al.* (2015)	Admiro	15-day old	11.61	11.61	11.61	174±29[4]	227±29	177±251
		25-day old	13.39	13.38	13.38	145±25	167±45	156±160
	Doloress	15-day old	11.92	11.92	11.92	188±31	249±23	177±296
		25-day old	10.61	10.61	10.61	131±31	167±29	167±212
	Growdena	15-day old	11.97	11.97	11.97	136±23	169±17	163±165
		25-day old	11.21	11.21	11.20	124±51	239±146	119±359
Data of Ho *et al.* (2016)	Admiro	Upper	8.61	8.61	8.61	120±16	156±17	128±180
		Lower	6.96	6.96	6.96	70±7	82±10	92±64
	Doloress	Upper	9.21	9.21	9.21	99±12	175±6	117±140
		Lower	8.27	8.28	8.28	114±8	129±9	118±140
	Growdena	Upper	8.15	8.15	8.15	114±8	146±16	120±172
		Lower	7.81	7.81	7.81	94±5	115±11	110±134

[1] Inner: This column contains the estimates of T_p and V_{cmax} for the scenario that assumed release of CO_2 from (photo)respiration in the inner cytosol.

[2] Gaps: This column contains the estimates T_p and V_{cmax} for the scenario that assumed release of CO_2 from (photo)respiration the cytosol gaps between chloroplasts.

[3] Outer: This column contains the estimates T_p and V_{cmax} for the scenario that assumed release of CO_2 from (photo)respiration in the outer cytosol.

[4] Estimated value of V_{cmax} ± standard deviation.

Table S4: ΔAIC for different cultivars (Admiro, Doloress, Growdena), leaf ages (15 days or 25 days after appearance), and scenarios (release of CO_2 from (photo)respiration in inner cytosol, cytosol gaps or outer cytosol). Experimental data were from the Berghuijs *et al.* (2015) data set

Cultivar	Leaf age (d)	Curve type	C_a (Pa)	O (kPa)	I_{inc} (μmol m^{-2} s^{-1})	ΔAIC		
						Inner[2]	Gaps[3]	Outer[4]
Admiro	15	$A - I_{inc}$	40	21	var	**0**[5]	9.43	57.7
		$A - C_a$	var[1]	21	1500	**0**	5.47	32.3
		$A - I_{inc}$	100	2	var	**0.18**	**0.12**	**0**
		$A - C_a$	var	2	1500	**0.49**	**0**	13.1
	25	$A - I_{inc}$	40	21	var	**0.42**	**0**	43.04
		$A - C_a$	var	21	1500	3.72	**0**	9.48
		$A - I_{inc}$	100	2	var	**0.37**	**0.25**	**0**
		$A - C_a$	var	2	1500	**1.55**	**0**	4.11
Doloress	15	$A - I_{inc}$	40	21	var	**0**	10.01	45.47
		$A - C_a$	var	21	1500	**0**	7.85	42.31
		$A - I_{inc}$	100	2	var	**0.05**	**0.03**	**0**
		$A - C_a$	var	2	1500	**0.27**	**0**	14.14
	25	$A - I_{inc}$	40	21	var	**0**	7.05	26.29
		$A - C_a$	var	21	1500	**0**	**1.96**	7.48
		$A - I_{inc}$	100	2	var	**0**	**0.03**	**0.09**
		$A - C_a$	var	2	1500	**0**	**0.10**	**1.09**
Growdena	15	$A - I_{inc}$	40	21	var	**0**	7.00	27.06
		$A - C_a$	var	21	1500	**0**	4.29	18.14
		$A - I_{inc}$	100	2	var	**0**	**0.02**	**0.06**
		$A - C_a$	var	2	1500	3.23	**0**	6.64
	25	$A - I_{inc}$	40	21	var	**0**	2.51	13.62
		$A - C_a$	var	21	1500	**0**	**1.81**	7.28
		$A - I_{inc}$	100	2	var	**0**	**0.02**	**0.06**
		$A - C_a$	var	2	1500	**1.09**	**0**	5.31

[1] Variable; during the measurement of a response curve either C_a or I_{inc} was varied, while the other variable was kept constant.

[2] Inner: This column contains the ΔAIC values for the scenario that assumed release of CO_2 from (photo)respiration in the inner cytosol.

[3] Gaps: This column contains the ΔAIC values for the scenario that assumed release of CO_2 from (photo)respiration the cytosol gaps between chloroplasts.

[4] Outer: This column contains the ΔAIC values for the scenario that assumed release of CO_2 from (photo)respiration in the outer cytosol.

[5] Bold values indicate that the corresponding model is either the best one from the three models ($\Delta AIC = 0$) or has substantial support relative to the best one ($0 < \Delta AIC \leq 2$).

Table S5: ΔAIC for different cultivars (Admiro, Doloress, Growdena), leaf layers (upper leaf and lower leaf), and scenarios (release of CO_2 from (photo)respiration in inner cytosol, cytosol gaps or outer cytosol). Experimental data were from the (Ho *et al.*, 2016) data set

Cultivar	Leaf type	Curve type	C_a (Pa)	O (kPa)	I_{inc} (μmol m^{-2} s^{-1})	ΔAIC		
						Inner[2]	Gaps[3]	Outer[4]
Admiro	Upper	$A - I_{inc}$	38	21	var	**0**[5]	5.86	33.78
		$A - C_a$	var[1]	21	1000	**0**	3.09	9.63
		$A - I_{inc}$	100	2	var	**0**	**0.00**	**0.00**
		$A - C_a$	var	2	1000	**0.04**	**0**	**1.68**
	Lower	$A - I_{inc}$	38	21	var	**0.04**	**0**	**0.705**
		$A - C_a$	var	21	1000	3.26	**1.49**	**0**
		$A - I_{inc}$	100	2	var	**0.05**	**0.00**	**0**
		$A - C_a$	var	2	1000	**1.03**	**1.20**	**0**
Doloress	Upper	$A - I_{inc}$	38	21	var	**0**	5.39	19.65
		$A - C_a$	var	21	1000	**0**	13.8	68.71
		$A - I_{inc}$	100	2	var	**0.03**	**0.02**	**0**
		$A - C_a$	var	2	1000	**0**	**0.71**	3.49
	Lower	$A - I_{inc}$	38	21	var	**0**	2.35	8.11
		$A - C_a$	var	21	1000	**0**	2.17	11.31
		$A - I_{inc}$	100	2	var	**0**	**0.01**	**0.02**
		$A - C_a$	var	2	1000	**0**	**0.06**	**0.22**
Growdena	Upper	$A - I_{inc}$	38	21	var	**0**	2.91	10.49
		$A - C_a$	var	21	1000	**0**	3.24	18.12
		$A - I_{inc}$	100	2	var	**0.03**	**0.02**	**0**
		$A - C_a$	var	2	1000	**0.01**	**0**	**0.27**
	Lower	$A - I_{inc}$	38	21	var	**0**	**0.75**	2.48
		$A - C_a$	var	21	1000	**0**	**0.59**	2.45
		$A - I_{inc}$	100	2	var	**0.03**	**0.02**	**0**
		$A - C_a$	var	2	1000	**0.02**	**0**	**0.02**

[1] Variable; during the measurement of a response curve either C_a or I_{inc} was varied, while the other variable was kept constant.

[2] Inner: This column contains the ΔAIC values for the scenario that assumed release of CO_2 from (photo)respiration in the inner cytosol.

[3] Gaps: This column contains the ΔAIC values for the scenario that assumed release of CO_2 from (photo)respiration the cytosol gaps between chloroplasts.

[4] Inner: This column contains the ΔAIC values for the scenario that assumed release of CO_2 from (photo)respiration in the outer cytosol.

[5] Bold values indicate that the corresponding model is either the best one from the three models (ΔAIC $= 0$) or has substantial support relative to the best one ($0 <$ ΔAIC ≤ 2).
